# Refined Interpretation of the Pistillate Flower in *Ceratophyllum* Sheds Fresh Light on Gynoecium Evolution in Angiosperms

**DOI:** 10.3389/fcell.2022.868352

**Published:** 2022-04-28

**Authors:** Dmitry D. Sokoloff, Elena S. El, Elena V. Pechenyuk, Laetitia Carrive, Sophie Nadot, Paula J. Rudall, Margarita V. Remizowa

**Affiliations:** ^1^ Biological Faculty, Lomonosov Moscow State University, Moscow, Russia; ^2^ Khoper State Nature Reserve, Varvarino, Russia; ^3^ Université Paris-Saclay, CNRS, AgroParisTech, Écologie, Systématique et Évolution, Orsay, France; ^4^ Royal Botanic Gardens, Kew, Richmond, United Kingdom

**Keywords:** apocarpy, Ceratophyllales, Chloranthales, congenital fusion, fossils, mesangiosperms, pseudomonomery, syncarpy

## Abstract

Molecular phylogenetic analyses have revealed a superclade of mesangiosperms with five extant lineages: monocots, eudicots, magnoliids, *Ceratophyllum* and Chloranthaceae. Both *Ceratophyllum* and Chloranthaceae are ancient lineages with a long fossil record; their precise placement within mesangiosperms is uncertain. Morphological studies have suggested that they form a clade together with some Cretaceous fossils, including *Canrightia*, *Montsechia* and *Pseudoasterophyllites*. Apart from *Canrightia*, members of this clade share unilocular gynoecia commonly interpreted as monomerous with ascidiate carpels. Alternatively, the gynoecium of *Ceratophyllum* has also been interpreted as syncarpous with a single fertile carpel (pseudomonomerous). We investigate patterns of morphological, anatomical and developmental variation in gynoecia of three *Ceratophyllum* species to explore the controversial interpretation of its gynoecium as either monomerous or pseudomonomerous. We use an angiosperm-wide morphological data set and contrasting tree topologies to estimate the ancestral gynoecium type in both *Ceratophyllum* and mesangiosperms. Gynoecia of all three *Ceratophyllum* species possess a small (sometimes vestigial) glandular appendage on the abaxial side and an occasionally bifurcating apex. The ovary is usually unilocular with two procambium strands, but sometimes bilocular and/or with three strands in *C. demersum*. None of the possible phylogenetic placements strongly suggest apocarpy in the stem lineage of *Ceratophyllum*. Rescoring *Ceratophyllum* as having two united carpels affects broader-scale reconstructions of the ancestral gynoecium in mesangiosperms. Our interpretation of the glandular appendage as a tepal or staminode homologue makes the *Ceratophyllum* ovary inferior, thus resembling (semi)inferior ovaries of most Chloranthaceae and potentially related fossils *Canrightia* and *Zlatkocarpus*. The entire structure of the flower of *Ceratophyllum* suggests strong reduction following a long and complex evolutionary history. The widely accepted notion that apocarpy is ancestral in mesangiosperms (and angiosperms) lacks robust support, regardless of which modes of carpel fusion are considered. Our study highlights the crucial importance of incorporating fossils into large-scale analyses to understand character evolution.

## Introduction

A key character of angiosperms is the presence of a gynoecium that consists of one or more carpels, each enclosing one or more protected ovules. There is widespread consensus that the free-carpellate (apocarpous) gynoecium is plesiomorphic in angiosperms, whereas the condition with fused carpels (syncarpy) is homoplastic and originated repeatedly during the course of angiosperm evolution (e.g., Doyle and Endress, 2000; Sauquet et al., 2017). Repeated gains of syncarpy have been shown to be of adaptive value, especially in allowing efficient competition among male gametophytes growing in different carpels, which can increase the fitness of the offspring (Endress, 1982; Armbruster et al., 2002). Yet our knowledge of the evolution of syncarpy in angiosperms is far from complete. Among mesangiosperms, a clade that includes all angiosperms except the early-divergent ANA-grade lineages (Moore et al., 2007), reconstructions of the ancestral gynoecium as either apocarpous or syncarpous are often equally parsimonious (e.g., Sokoloff et al., 2013a; Massoni, 2014). Thus, ironically, even the iconic gynoecium of *Magnolia* could be secondarily free-carpellate.

**FIGURE 1:**
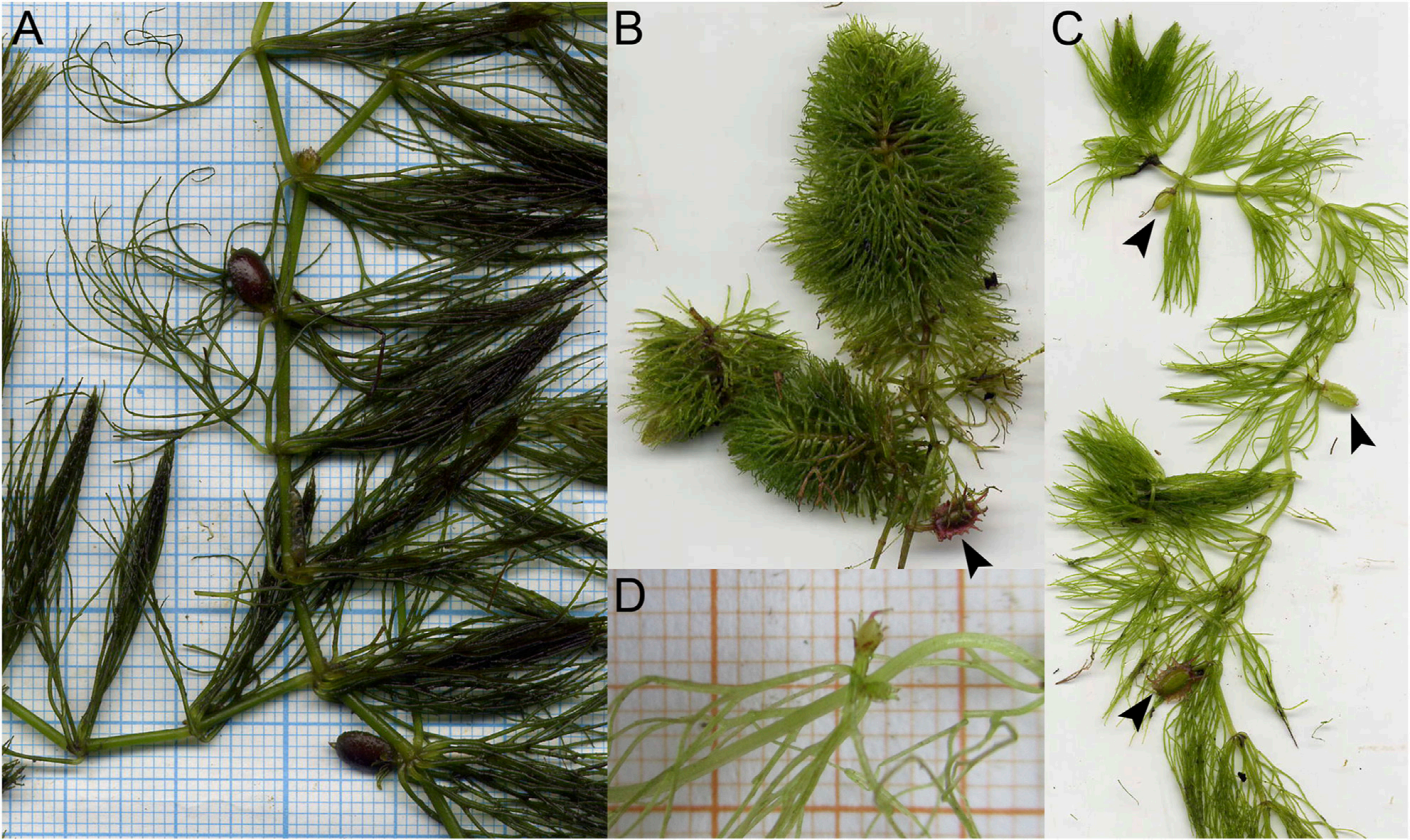
Living plants of *Ceratophyllum* spp. in Khoper State Nature Reserve, Russia (photos: E.V. Pechenyuk). **(A)** *Ceratophyllum submersum*, portion of plant with two fruits, both sessile and smooth. **(B)** Terrestrial plant of *C. tanaiticum* with a stalked, spinose fruit (arrowhead). **(C)** Aquatic plant of *C. tanaiticum* with three fruits at successive developmental stages (arrowheads). Fruit spines appear late in development. **(D)** Postanthetic pistillate flower of *C. tanaiticum*.

Accurate scoring of each terminal group ultimately depends on morphological interpretation and is crucial for analyses of character evolution, especially for taxonomically isolated and ancient lineages. At the same time, such interpretation can be highly problematic, as in the gynoecium of *Ceratophyllum* (Figures 1A–C), an ancient aquatic genus of about six species with minute unisexual flowers and peculiar underwater pollination (Les, 1986; Szalontai et al., 2018). Currently recognized as a distinct order, Ceratophyllales, *Ceratophyllum* has been described as a ‘rogue’ or ‘orphan’ taxon because of its problematic position in phylogenetic analyses (One Thousand Plant Transcriptomes Initiative, 2019; Albert and Renner, 2020). Consistent with the aquatic habit, the vegetative body of *Ceratophyllum* lacks stomata or roots at any stage of development; it also lacks xylem conducting elements. The characteristic “leaves” are dichotomously divided 1–4 times with linear denticulate segments; they appear whorled (verticillate), but could plausibly be interpreted as decussate, with several leaf-like organs at each node (Schaeppi, 1935; Iwamoto et al., 2015). The fruits are one-seeded, often with characteristic spine-like appendages. Fossil fruits resembling those of extant *Ceratophyllum* associated with dichotomous leaves have been found in Early Cretaceous deposits (Albian of Kansas, United States, Dilcher and Wang, 2009; Wang and Dilcher, 2018).

To explore the controversial interpretation of the gynoecium in *Ceratophyllum* as either monomerous (having a single carpel) or pseudomonomerous (superficially resembling the monomerous condition, but actually composed of at least two fused carpels), we investigated morphological variation in pistillate flowers of three species, *C. demersum* L., *C. submersum* L. and *C. tanaiticum* Sapjegin, which represent three of the four major clades within *Ceratophyllum* identified by molecular data (Szalontai et al., 2018). Until now, all studies of flower development in *Ceratophyllum* using scanning electron microscopy (SEM) have concerned a single species, *C. demersum*. Mavrodiev et al. (2021) proposed segregation of the North American species *C. echinatum* A. Gray into a new and presumably monotypic genus *Fassettia*, but material of this species was not available for the present study.

Historically, *Ceratophyllum* was regarded as a specialized relative of the water-lily families Nymphaeaceae and Cabombaceae (e.g., Cronquist, 1981; Takhtajan, 1987), partly because the dissected submerged leaves of *Cabomba* resemble those of *Ceratophyllum* (Schaeppi, 1935). However, molecular phylogenetic analyses do not support a sister-group relationship between *Ceratophyllum* and water lilies (including Hydatellaceae) and detailed studies of embryology and seed anatomy have revealed strong morphological differences between them.

**FIGURE 2:**
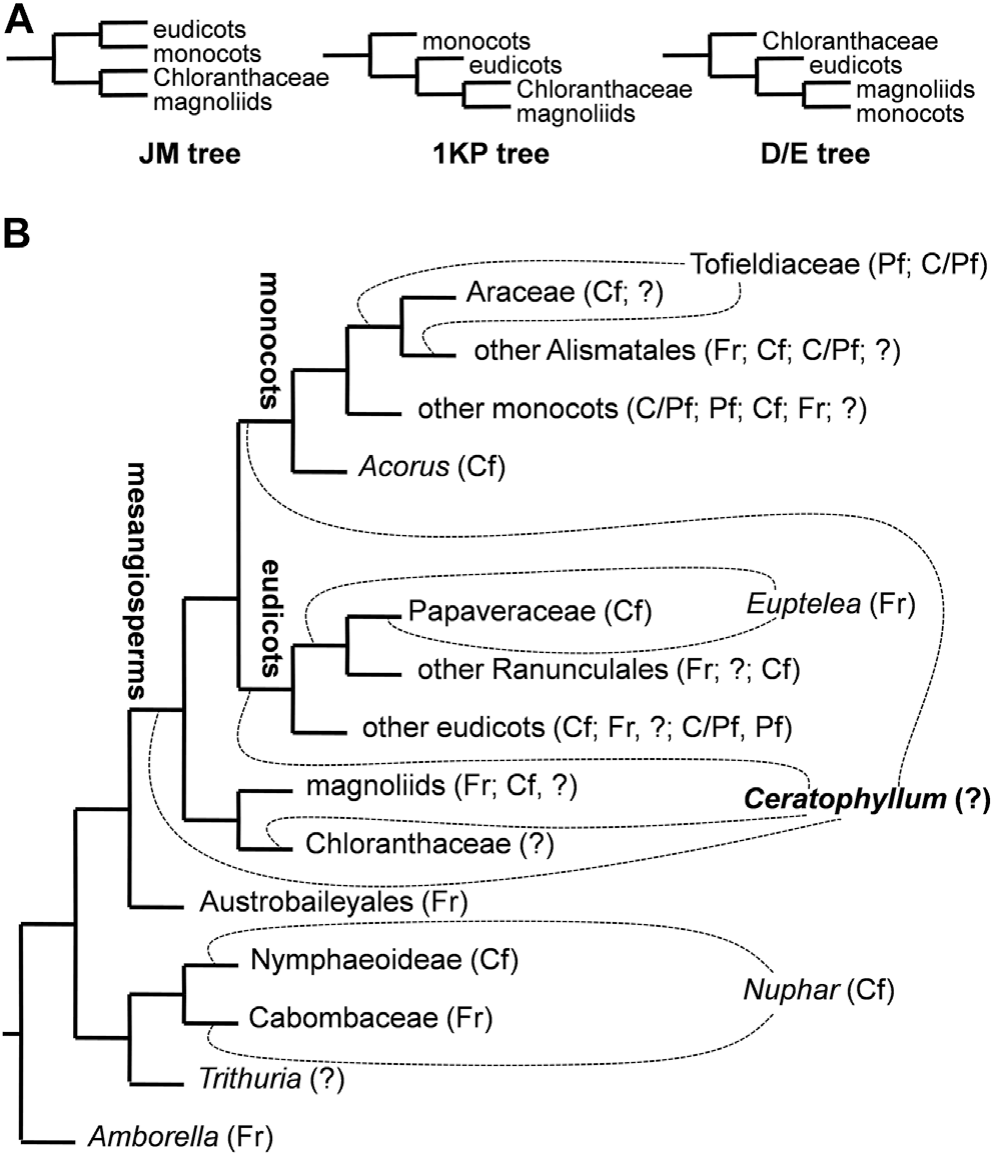
Ambiguities among various phylogenetic trees of angiosperms considered in the present study. **(A)** Simplified diagrams of three major hypotheses of relationships among clades of mesangiosperms with the position of *Ceratophyllum* omitted. See text for details on naming these topologies. **(B)** simplified ‘main’ tree topology (JM tree) explored in an important recent study of character evolution in angiosperms (Sauquet et al., 2017). Dotted lines indicate contrasting positions for *Ceratophyllum* and three other problematic taxa. Abbreviations after taxon names indicate types of fusion between carpels in a rough sequence of their frequency/closeness to the root in each terminal group: Fr, carpels free; Cf, congenital fusion: Pf, postgenital fusion: C/Pf, both types of fusion in the same gynoecium: ? gynoecium monomerous or reportedly monomerous and thus the fusion character is apparently not applicable.

The earliest molecular phylogenetic studies, based on the *rbcL* coding region, supported a morphology-based hypothesis of Les (1988) in placing *Ceratophyllum* on a long branch as sister to all other extant angiosperms (Les et al., 1991; Chase et al., 1993). This topology was challenged as the number of available molecular phylogenetic markers increased, instead supporting an isolated position for *Ceratophyllum* among early-diverging lineages of angiosperms. Subsequently, *Ceratophyllum* has been placed in various phylogenetic positions depending on the markers and methods used (Figure 2), most commonly either as sister to eudicots (Jansen et al., 2007; Moore et al., 2007; One Thousand Plant Transcriptomes Initiative, 2019; Guo et al., 2021) or as sister to Chloranthaceae, another ancient lineage with relatively few extant taxa (Antonov et al., 2000; Moore et al., 2011; Zeng et al., 2014; Xue et al., 2020). However, there is often poor statistical support for phylogenetic placement in either location, even in some analyses that include considerable genomic data (Gitzendanner et al., 2018; One Thousand Plant Transcriptomes Initiative, 2019; Yang et al., 2020). Furthermore, *Ceratophyllum* not only lacks the gamma whole-genome triplication (WGT) that characterises most (though not all) members of the eudicot lineage; it also lacks the whole-genome duplications (WGDs) of magnoliids, and possesses some independent ancient WGDs (Albert and Renner, 2020; Yang et al., 2020).

Endress and Doyle (2009), Endress and Doyle (2015), Doyle and Endress (2018) summarized the morphological evidence for placement of Ceratophyllaceae as sister to Chloranthaceae. These two families share some similarities, such as pendent orthotropous ovules, the presence of a single ovule per ovary, non-spiral phyllotaxis of vegetative shoots (decussate in Chloranthaceae and pseudo-verticillate in Ceratophyllaceae, modified from decussate). However, they differ from each other in both habit and ecology, as Chloranthaceae are terrestrial herbs and shrubs. The staminate reproductive structures of *Ceratophyllum* resemble those of *Hedyosmum* (Chloranthaceae). The pistillate structures of *Ceratophyllum* possess a proximal whorl of sterile appendages that are sometimes interpreted as sepals (Cronquist, 1981; Takhtajan, 1987), but most likely represent an extrafloral involucre (Les, 1986; Endress, 1994; Endress, 2001; Endress and Doyle, 2009; Endress and Doyle, 2015); the remaining part resembles the pistillate flower of *Ascarina* (Chloranthaceae), which consists of a single ascidiate carpel (Les, 1986; Endress, 1994; Endress, 2001). Detailed phylogenetic studies of Cretaceous fossils that are putatively related to Chloranthaceae and *Ceratophyllum* have concluded that extant Chloranthaceae and *Ceratophyllum* belong to a clade that was more diverse during early phases of angiosperm evolution, both ecologically and morphologically (Doyle and Endress, 2014; Kvaček et al., 2016; Doyle and Endress, 2018; Gomez et al., 2020).

Our study presents new data on developmental morphology and anatomy in *Ceratophyllum* to evaluate organ homologies in the genus. We employ a “back to basics” approach that revisits and deconstructs perceived anomalies in gynoecium structure in *Ceratophyllum* and tests whether available evidence can help to explain them. New discoveries in genomics and new fossils offer exciting opportunities for understanding early angiosperm evolution. However, testing evolutionary hypotheses is only possible using accurate homology assessments. Our goals are 1) to determine whether comparative developmental morphology and phylogenetic placement can clarify interpretation of the gynoecium in *Ceratophyllum*; and 2) infer whether contrasting interpretations within this unusual and isolated genus could influence broader reconstruction of gynoecium evolution in angiosperms in general*.*

## Materials and Methods

Whole plants and young shoot tips of three species of *Ceratophyllum* (*C. demersum, C. submersum* and *C. tanaiticum*) were fixed in 70% ethanol. Material of *C. demersum* was collected from a pond in Zvenigorod Biological Station of Moscow State University (Odintsovsky distr., Moscow Prov.; voucher: *Sokoloff & Remizowa s.n.*, 2016, MW). Material of *C. tanaiticum* and *C. submersum* was collected in Khoper State Nature Reserve, Voronezh prov., Russia (vouchers: *Pechenyuk 16*, 2012; *Pechenyuk 32*, 2017, MW).

**FIGURE 3:**
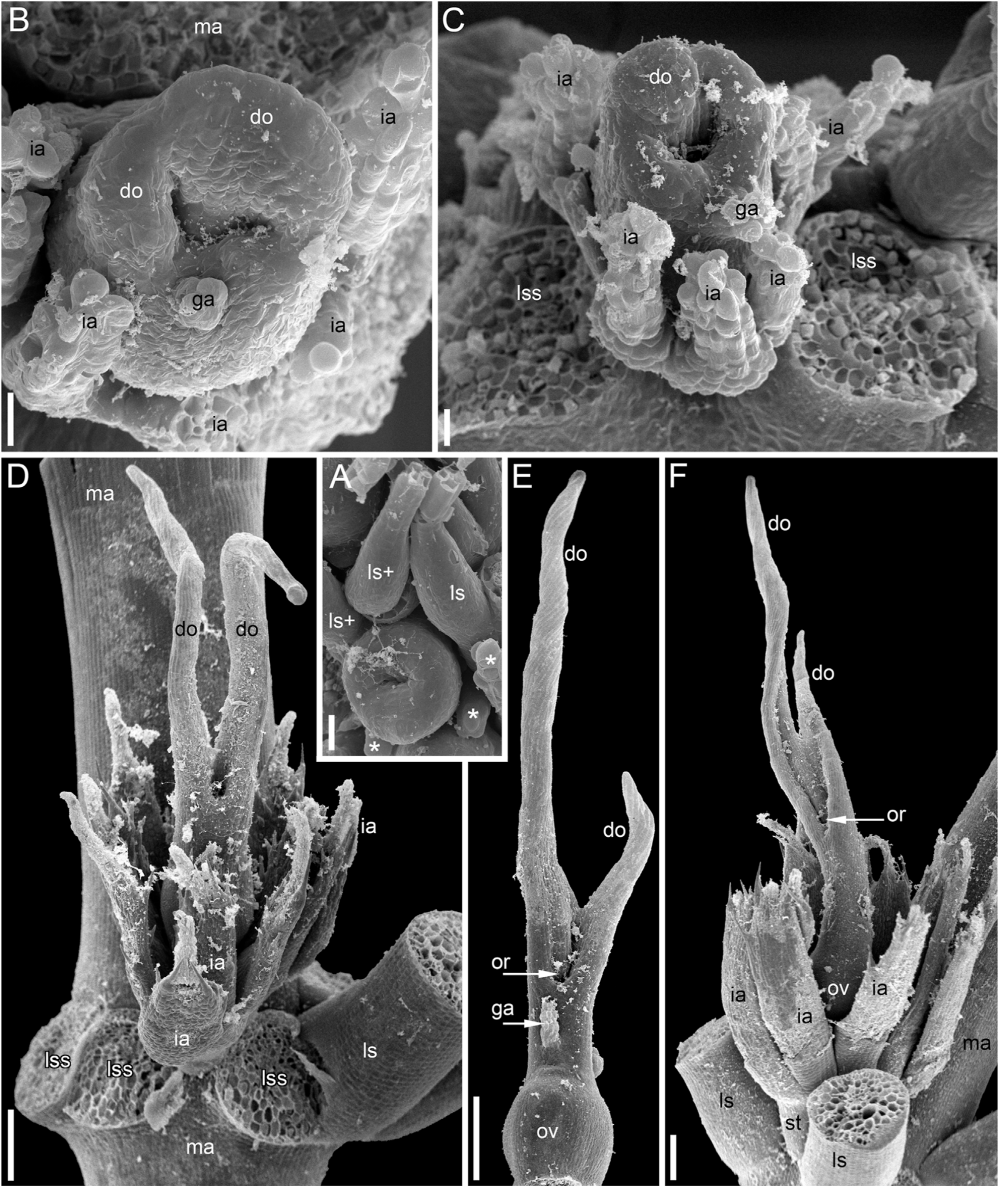
Pistillate flowers of *Ceratophyllum demersum* (SEM). **(A)** Very young flower with involucral appendages of the reproductive unit and any gynoecium appendages still inconspicuous. Adaxial side up. Small organs near the flower (asterisks) are leafy segments of a vegetative branch situated below the flower. **(B,C)** Young flowers, adaxial side up. The glandular gynoecial appendage is already well-developed, but distal outgrowth(s) are still very short. **(B)** Gynoecium developing a bilobed distal outgrowth or two distal outgrowths, a wider one adaxial and a narrower one left transversal; the gland is abaxial. **(C)** The gland and a young distal outgrowth are at the opposite radii, which are oblique relative to the median plane of the flower. Note that the flower is extra-axillary, located between two vegetative leafy segments of a node. **(D)** Abaxial view of a flower with two equal distal gynoecial outgrowths. The flower is attached to a stem node between two leafy segments that are excised. Sections of this flower are shown in Figure 7. **(E)** Abaxial view of flower with involucral appendages removed. The two distal appendages are of unequal size. **(F)** Side view of flower with unequally bilobed distal appendage. Scale bars = 30 μm in **(A–C)**, 300 μm in **(D–F)**.

For Scanning Electron Microscopy (SEM), material was dissected in 70% ethanol and transferred to 100% acetone using the following series: 96% ethanol (twice for 30 min), 96% ethanol: 100% acetone (1:1 v/v, 30 min), 100% acetone (three times for 30 min). About ten individuals and more than 30 flowers of each species were examined. The material was critical-point dried using a Hitachi HCP-2 critical-point dryer (Hitachi, Japan), then coated with gold and palladium using a Eiko IB-3 ion-coater (Tokyo, Japan) and observed using a CamScan S-2 (Cambridge Instruments, London, United Kingdom) at the Laboratory of Electron Microscopy at the Biological Faculty of Moscow University. Some flowers were sectioned anatomically using standard anatomical methods, including paraplast embedding and serial sectioning at a thickness of 15 μm using an HM 355S Automatic Microtome (Thermo Fisher Scientific, Waltham, MA, United States). The sections were stained in picroindigocarmine and carbolic fuchsine using a Varistain GEMINI ES Automated Slide Stainer (Thermo Fisher Scientific, Waltham, MA, United States) and mounted in Biomount. Images were taken using a Zeiss (Göttingen, Germany) Axioplan microscope. One flower was sectioned in Technovit (Heraeus-Kulzer, Wehrheim, Germany) 7,100 after SEM imaging (Figure 3C).

Two strategies were employed to reconstruct the evolution of gynoecium morphology, coded here as a binary character (carpels free/carpels fused). Ancestral state reconstruction was performed with parsimony using Mesquite v.3.6 (Maddison and Maddison, 2018).

1) To explore whether placement in a phylogenetic context helps in disentangling gynoecium morphology of *Ceratophyllum*, we used the angiosperm-wide data set of Sauquet et al. (2017), where carpel fusion is scored as uncertain in *Ceratophyllum.* Ancestral states were reconstructed using parsimony on various tree topologies (see below), recording the character state inferred for the node from which this taxon diverged.
2) To explore whether reconstructions of gynoecium evolution in angiosperms are sensitive to contrasting interpretations of the pistillate flower in *Ceratophyllum*, various tree topologies (see below) were investigated using the same data set, with the only exception that the *Ceratophyllum* gynoecium was scored as syncarpous (pseudomonomerous) in accordance with our morphological observations. The ancestral condition inferred for mesangiosperms was recorded for each tree topology. We focused on mesangiosperms because *Ceratophyllum* represents one of the five well-supported clades composing mesangiosperms; its relationships with the four other clades (eudicots, monocots, magnoliids and Chloranthaceae) remain insufficiently resolved.

Sauquet et al. (2017) did not differentiate between gynoecia with congenital and postgenital carpel fusion, which differ fundamentally in their development (Endress, 2006; Sokoloff et al., 2018b). Congenital and postgenital fusions are apparently governed by different gene regulatory networks. The extensively studied *NAM/CUC3* genes (Phillips et al., 2020) are specifically related to congenital fusions. Strictly speaking, only gynoecia with congenital fusion between carpels are syncarpous. Lumping together different types of fusion would favour reconstructions with early gain(s) of syncarpy. To explore this potential effect, we edited the original character 403_A (fusion of ovaries, binary) of Sauquet et al. (2017) to retain only instances of angiosperms with congenital fusion between carpels (syncarpy in the narrow sense). In agreement with Sauquet et al. (2017), we considered that fusion is present when the carpels are united for more than 5% of their length (though a more careful approach is sometimes needed, as in Betulaceae, when free styles/stigmas are much longer than the ovary at anthesis). The edited data set is available as an online supplement to this paper (Supplementary File 1). As highlighted by Sauquet et al. (2017), accurate developmental data could not be collected for all of the 792 species included in this data set, so in some cases we used indirect evidence or data on taxonomically related species to interpolate missing developmental data. Thus, our edited character is not precisely documented for all species. However, our goal is to explore what kind of effects such rescoring may have, rather than finding an ultimate and correct reconstruction. In analyses related to our strategies 1) and (2), we used both the original and edited (congenital) character of fusion between carpels. To summarize the complex terminology, we note that if the gynoecium of *Ceratophyllum* is interpreted as pseudomonomerous, then it is syncarpous, consisting of congenitally fused carpels. If the gynoecium is interpreted as monomerous, then it cannot be scored in the data set as either possessing or lacking fusion between carpels, because there is only one carpel (data are considered as missing).

All ancestral state reconstructions were performed using one and the same set of 96 tree topologies selected in such a way as to capture the most important areas of instability in nodes that are close to the node of mesangiosperms (Figure 2; Table 1). We considered three more commonly discussed hypotheses of relationships among the species-rich clades of mesangiosperms (Figure 2A). Other possible hypotheses (Zeng et al., 2014; Guo et al., 2021) differ only in position of Chloranthaceae, a group for which the characters discussed here should be scored as uncertain. The three topologies considered here merit special naming (Figure 2A). The DE and JM trees were broadly designated by Endress and Doyle (2009) because the former was found in studies by Jansen et al. (2007) and Moore et al. (2007) and the latter by Doyle and Endress (2000). The 1,000 transcriptome (1 KP) tree was revealed by the One Thousand Plant Transcriptomes Initiative (2019). The DE, JM and 1 KP topologies cover all logically possible relative placements of eudicots, monocots and magnoliids (Figure 2A). For each of these three relationships among the species-rich mesangiosperm clades, all combinations of the possible positions of *Ceratophyllum* plus three other phylogenetically pivotal taxa, *Nuphar*, *Euptelea* and Tofieldiaceae (Figure 2B), were examined systematically. *Nuphar* is traditionally regarded as sister to other Nymphaeaceae (Nymphaeoideae), with syncarpy being a major difference of the family from its sister group, Cabombaceae (Les et al., 1999; Borsch et al., 2008). Recent data based on sparse but representative taxon sampling show that *Nuphar* could be instead sister to Cabombaceae (Gruenstaeudl, 2019). The occurrence of carpel fusion in the third family of Nymphaeales, Hydatellaceae (*Trithuria*), is uncertain because each unicarpellate pistil in reproductive units of *Trithuria* could represent an individual reduced flower (Rudall et al., 2007). Thus, reconstruction of the ancestral gynoecium condition in a clade of all angiosperms except *Amborella* is directly sensitive to the unstable placement of *Nuphar*. Two families–the apocarpous Eupteleaceae (single genus *Euptelea*) and the syncarpous Papaveraceae–are unresolved at the root of the basal eudicot lineage Ranunculales (Carrive et al., 2020). Similarly, two families with contrasting gynoecium morphology (Tofieldiaceae and Araceae) are unresolved at the root of Alismatales, a lineage of basal monocots (Luo et al., 2016; Ross et al., 2016; Givnish et al., 2018). All nodes not discussed above were retained as in the preferred tree topology used by Sauquet et al. (2017).

**TABLE 1:** Ancestral state reconstructions with parsimony for carpel fusion in a stem-group lineage of *Ceratophyllum* on three different major phylogenetic topologies (JM, 1 KP or DE) combined with all possible positions of *Ceratophyllum*, *Nuphar*, *Euptelea* and Tofieldiaceae (Figure 2), showing to what extent phylogenetic placement aids homology reconstruction. Each cell shows reconstructed conditions for *Ceratophyllum* (to which no a priori state was assigned) in three contrasting topologies using the original (any fusion)/edited (congenital fusion) data from Sauquet et al. (2017). P = fusion between carpels present; U = presence or absence of fusion uncertain. Boldface indicates the conditions found in the eight topologies that are sensitive to the coding of syncarpy.

		*Euptelea* sister to all other Ranunculales		*Euptelea* sister to Papaveraceae	
		Araceae sister to all other Alismatales	Tofieldiaceae sister to all other Alismatales	Araceae sister to all other Alismatales	Tofieldiaceae sister to all other Alismatales
		JM	JM	JM	JM
		1 KP	1 KP	1 KP	1 KP
		DE	DE	DE	DE
*Ceratophyllum* sister to Chloranthaceae	*Nuphar* + Nymphaeoideae	U/U	U/U	U/U	U/U
		U/U	U/U	P/P	P/P
		U/U	U/U	U/U	U/U
	*Nuphar* + Cabombaceae	U/U	U/U	U/U	U/U
		U/U	U/U	P/P	P/P
		U/U	U/U	U/U	U/U
*Ceratophyllum* sister to eudicots	*Nuphar* + Nymphaeoideae	U/U	U/U	**P/U**	**P/U**
		U/U	U/U	P/P	P/P
		U/U	U/U	P/P	P/P
	*Nuphar* + Cabombaceae	U/U	U/U	**P/U**	**P/U**
		U/U	U/U	P/P	P/P
		U/U	U/U	P/P	P/P
*Ceratophyllum* sister to monocots	*Nuphar* + Nymphaeoideae	U/U	U/U	**P/U**	**P/U**
		U/U	U/U	P/P	P/P
		U/U	U/U	P/P	P/P
	*Nuphar* + Cabombaceae	U/U	U/U	**P/U**	**P/U**
		U/U	U/U	P/P	P/P
		U/U	U/U	P/P	P/P
*Ceratophyllum* sister to other mesangiosperms	*Nuphar* + Nymphaeoideae	U/U	U/U	U/U	U/U
		U/U	U/U	U/U	U/U
		U/U	U/U	U/U	U/U
	*Nuphar* + Cabombaceae	U/U	U/U	U/U	U/U
		U/U	U/U	U/U	U/U
		U/U	U/U	U/U	U/U

## Results

### Pistillate Flower Structure and Development in *Ceratophyllum demersum*

The pistillate flowers (Figures 3C,D,F) develop on shoot nodes between the radii of the bifurcating vegetative organs (leafy segments; “leaves” of taxonomic descriptions), whereas the vegetative branches are axillary.

**FIGURE 4:**
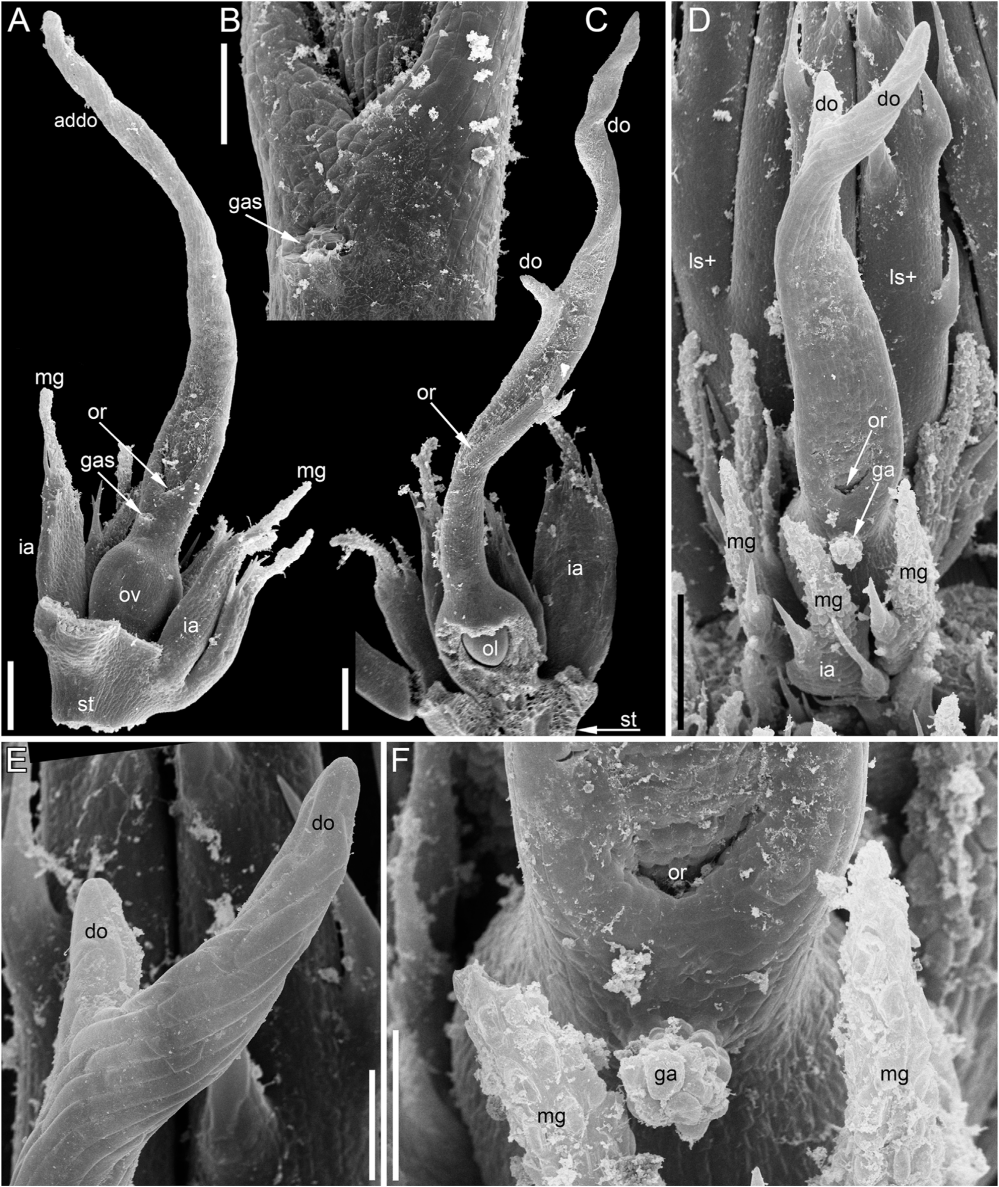
Pistillate flowers of *Ceratophyllum demersum* (SEM). **(A)** Abaxial view of flower with single, unlobed distal gynoecial outgrowth. Some involucral appendages are removed to show the ovary. The glandular appendage of the gynoecium is abscised, but its scar is visible. **(B)** Detail of **(A)** showing the gynoecial orifice and a scar of the ascised glandular appendage. **(C)** Dissected flower with unequally bilobed distal gynoecial outgrowth. The ovary is unilocular, with a single pendent ovule. **(D)** Abaxial view of flower with bilobed distal gynoecial outgrowth. **(E)** Detail of **(D)** showing the two lobes of the distal outgrowth. **(F)** Detail of **(D)** showing glandular gynoecial appendage. Scale bars = 300 μm in **(A,C,D)**, 100 μm in **(B,E,F)**.

**FIGURE 5:**
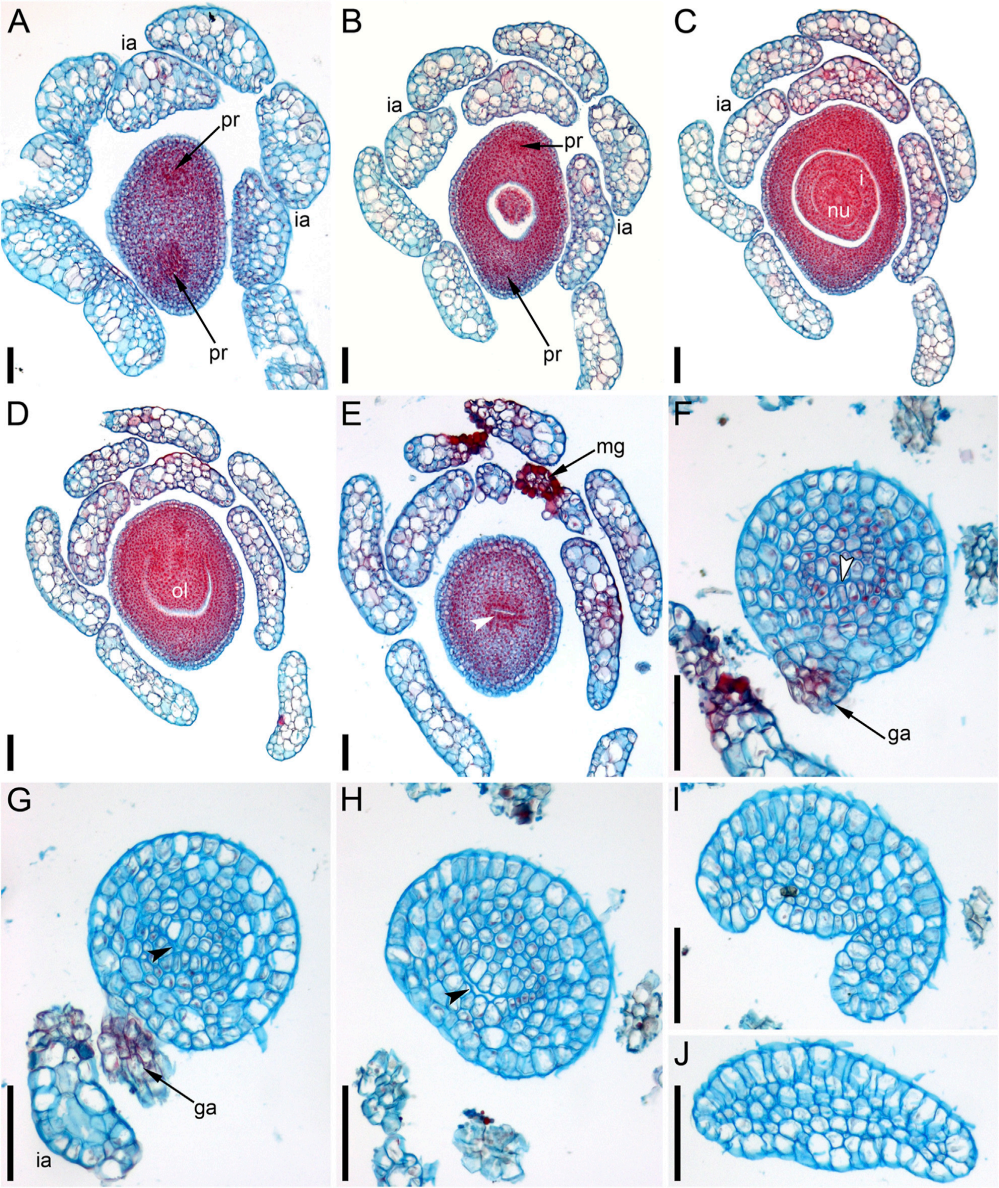
Ascending series of anatomical sections of pistillate flower of *Ceratophyllum demersum* with single, abaxial glandular gynoecial appendage (LM). The flower is similar to the one illustrated in Figure 4A. **(A)** Below the ovary locule, note two vascular bundles. **(B)** Close to the bottom of the ovule. **(C)** Middle part of the ovule. **(D)** Level of the ovule attachment. **(E–H)** Sections between the ovary locule and the gynoecial orifice (arrowheads, stylar canal). **(F,G)** Sections showing the glandular appendage. **(I,J)** Distal gynoecial outgrowth. Scale bars = 100 μm in **(A–J)**.

**FIGURE 6:**
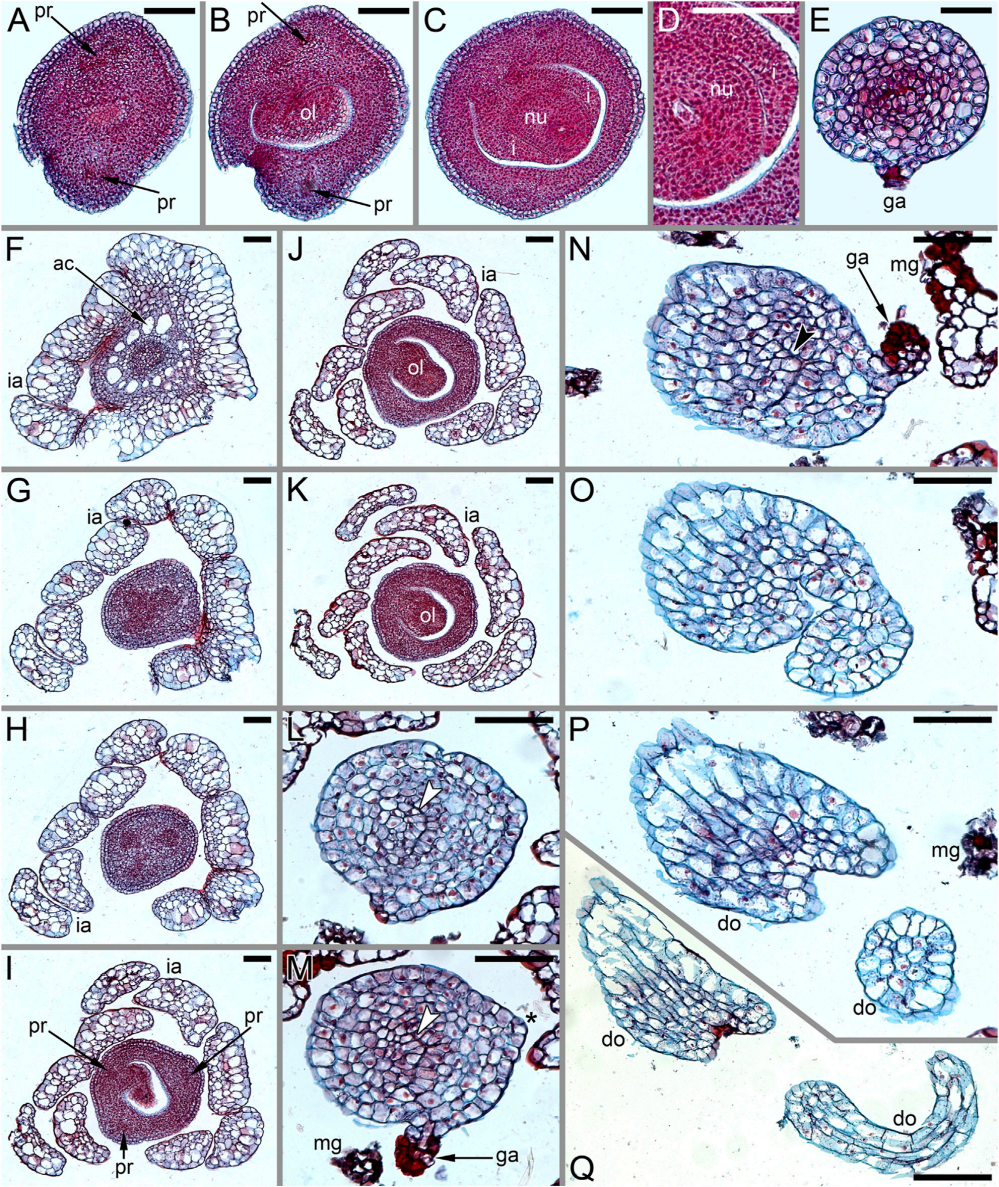
Anatomy of pistillate flowers of *Ceratophyllum demersum*, ascending series of transverse anatomical sections (LM). **(A–E)** Flower with single, abaxial glandular gynoecial appendage and single horizontally directed ovule (involucral appendages not shown). Ovule insertion is nonmedian as evidenced by its position relative to the two procambial strands and the glandular appendage. **(A)** Below the ovary locule. **(B–D)** Ovary locule and ovule. **(D)** Detail of section just above **(C)**; note the clearly visible boundary between the nucellus and the integument. **(E)** Level of glandular appendage. **(F–Q)** Flower with two glandular gynoecial appendages and a single horizontally directed ovule. **(F)** Level of attachment of involucral appendages. **(G)** Trilobed bundle in the gynoecium stalk. **(H)** Three distinct bundles in the gynoecium stalk. **(I–K)** The ovule is attached along almost the entire length of the ovary locule. **(L–N)** Sections between the ovary locule and the gynoecial orifice (arrowheads, stylar canal). **(M, N)** Sections showing the two glandular appendages. Each of these two sections shows only one glandular appendage, but these are two different appendages. Asterisk in (M) indicates the radius of the appendage visible in (N). **(O)** Gynoecium orifice. **(P, Q)** Distal outgrowths of the gynoecium. Scale bars = 100 μm in **(A–Q)**.

**FIGURE 7:**
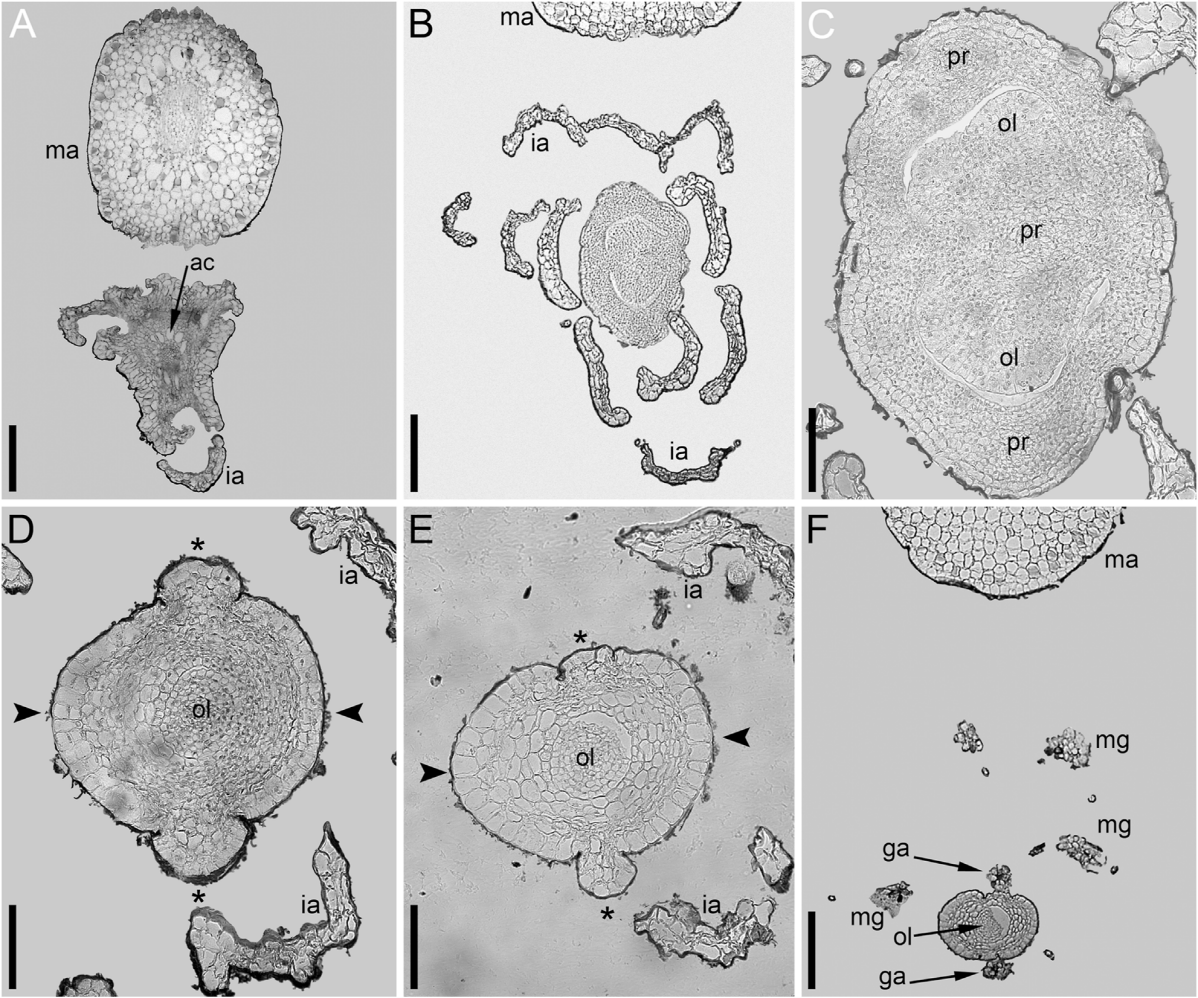
Ascending series of transverse anatomical sections of pistillate flower of *Ceratophyllum demersum* with two glandular gynoecial appendages and basally bilocular gynoecium (LM). A general view of this flower is shown in Figure 3D. The flower has been serially sectioned after taking SEM images. **(A)** Level of attachment of involucral appendages. **(B,C)** Bilocular region of the gynoecium. Each locule has an ovule. **(D–F)** Unilocular region of the gynoecium with the third ovule. Arrowheads in D,E show radii of attachment of the two long distal outgrowths (see Figure 3D). Asterisks in D,E show radii of the two glandular appendages that are visible in **(F)**. **(F)** Level of attachment of the two glandular appendages. Scale bars = 300 μm in **(A,B,F)**, 100 μm in **(C–E)**.

The pistillate flower has an involucre consisting of a whorl of leaf-like appendages, each bearing an apical mucilaginous gland and acute unicellular teeth on either side (Figures 3B–D–D,F, 4A,B). The term ‘pistillate reproductive unit’ can be used for a flower surrounded by an involucre. The number of involucral appendages is not precisely fixed, ranging from six to eleven in our material (Figures 3B–D–D, 5A–D–D, 6F–K–K 6F–K, 7B). Below the level of attachment of the involucral appendages there is a stalk, which is short but rather wide (Figures 3F, 4A,C). The stalk is triangular in cross-section, with one angle abaxial and two angles transversal-adaxial. Like stem internodes, it has a ring of large air canals whose distal parts can be seen in Figures 6F, 7A.

At the stage when the first evidence can be observed of a central depression in the pistil (the future gynoecium cavity), the involucral appendages of the flower are still inconspicuous and the leafy segments of the stem node are much longer than the flower (Figure 3A). At the youngest observed stage, the abaxial side of the gynoecium rim is wider than the adaxial side (Figure 3A). There is no evidence of a mucilaginous appendage at this stage. Here and below, we define the abaxial and adaxial side of the gynoecium relative to the main shoot axis, which is labelled ‘ma’ in Figures 3D,F. Strictly speaking, the pistillate flower belongs to a short specialized lateral shoot (=axis of the reproductive unit) with a whorl of involucral appendages followed by the flower. There is no way of assessing the orientation of the pistillate flower relative to the axis of the reproductive unit.

The next observed stage shows more extensive development of the adaxial side, where the rim of the gynoecium is either unequally bilobed (Figure 3B) or with a localized incipient distal outgrowth (Figure 3C). Involucral appendages are well-recognizable but slightly shorter than the gynoecium at this stage (Figures 3B,C). A glandular (mucilaginous) abaxial appendage is already present in the abaxial (Figure 3B) or almost abaxial (Figure 3C) position close to the rim of the gynoecium.

At anthesis, the abaxial glandular appendage is readily recognizable at a distance below the orifice of the gynoecium and well above the level of the attachment of the ovule (Figures 3E, 4A,B,D,F, 5F,G). In a few cases when the glandular appendage is abscised, its scar is clearly recognizable (Figures 4A,B). Here, we also provide the first documentation of sporadic gynoecia possessing two glandular appendages rather than the usual one (Figures 6M,N, 7F). The glandular appendages of the gynoecium (labelled ga) have the same structure as the mucilage gland (labelled mg) at the apex of the involucral appendage (Figure 6M), but they are shorter than the latter (Figure 4D). Note that the two glandular appendages of the gynoecium illustrated in Figures 6M,N are inserted at slightly different levels and thus one of them is visible in M and the other in N.

Some anthetic flowers possess a single large distal outgrowth on the adaxial side, in the same radius as the site of the ovule attachment (Figures 4A, 5I,J). The proximal part of the outgrowth is more or less flattened and its distal part is usually spirally twisted. Flowers with two equally long distally twisted outgrowths in symmetrical transversal positions (slightly shifted towards the adaxial side of the flower) are also found (Figure 3D), together with a range of gynoecia transitional between these two extremes (Figures 3E,F, 4C–E–E). Transitional forms show various degrees of unilateral fusion of the two outgrowths on the adaxial side of the flower, one of them often shorter than the other (Figures 3E,F, 4C,D).

In gynoecia with a single glandular (mucilaginous) appendage, the ovary is unilocular with a single ovule attached adaxially. The ovule is usually pendent, with the micropyle facing the base of the ovary (Figures 4C, 5B–D–D), but sometimes the ovule is oriented horizontally, with the micropyle facing the opposite side of the ovary (Figures 6B–D). The ovary wall has two procambial strands, one adaxial, entering the ovule, the other abaxial (Figures 5A–D–D, 6A–C–C). Both strands are no longer recognizable above the ovule attachment. The stylar canal is present as a narrow elliptical transversally elongate cavity in sections immediately above the ovule (Figure 5F) but postgenitally sealed for the rest of its length (Figures 5F,G). The line of the closure of the canal is straight or U-shaped in cross-sections.

Gynoecia with two mucilaginous appendages have three procambial strands. The gynoecium in Figures 6F–Q is unilocular and has a single ovule oriented horizontally. One procambial strand is in the ovule radius, two others are in the radii of the mucilaginous appendages (Figures 6H,I). A wide distal outgrowth is in the ovule radius and a narrow distal outgrowth is in the opposite radius (Figures 6P,Q). Figure 3E provides an external view of a gynoecium with two unequal distal outgrowths similar to that in Figures 6F–Q. Sections of the gynoecium illustrated in Figure 3D with two equal and transversally directed distal outgrowths (Figure 7) display two symmetrically arranged mucilaginous appendages, one adaxial and the other abaxial (Figure 7F). The proximal part of the ovary is bilocular, with one locule adaxial, the other abaxial (Figures 7B,C), each locule containing a horizontally oriented ovule (Figure 7C). The distal part of the ovary is unilocular and contains a third ovule (Figures 7D,E) that is oriented upwards and attached at the right side of the unilocular region, just above the transition from the bilocular part. Two procambial strands are located in the radii of the glandular appendages; a third strand in the septum between the locules serves all three ovules.

### Pistillate Flower Structure and Development in *Ceratophyllum tanaiticum*

**FIGURE 8:**
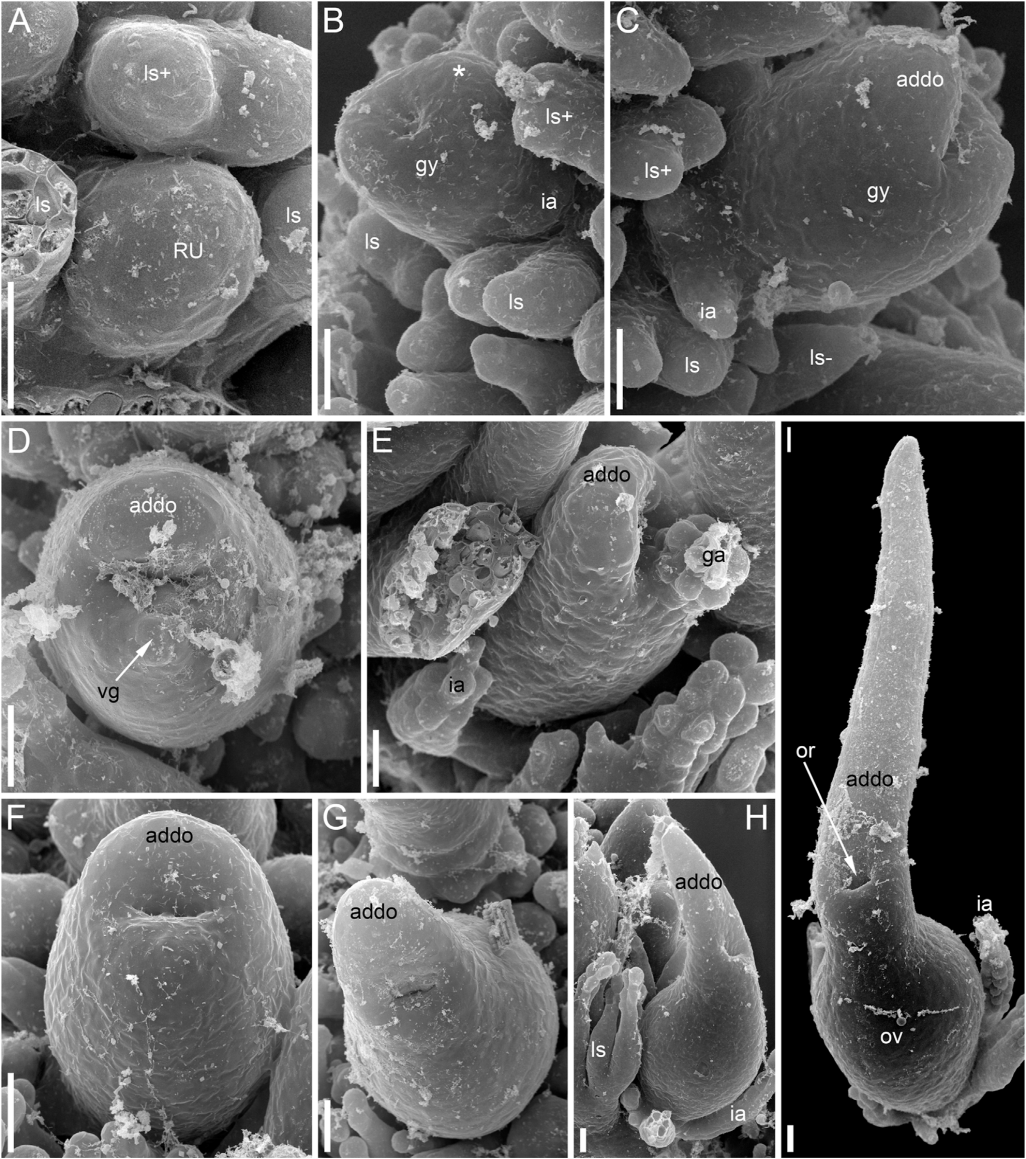
Pistillate flower development in *Ceratophyllum tanaiticum* (SEM). **(A)** Young apparently pistillate reproductive unit inserted between two leafy segments of a node. The bilobed leafy segment of the subsequent node is visible. Involucral appendages of the reproductive unit are not yet initiated. **(B)** Early stage of gynoecium development. The adaxial side of the gynoecium is indicated by asterisk. Involucral appendages are initiated. **(C)** Side view of a gynoecium with adaxial distal outgrowth initiated. Involucral appendages are still very short. **(D)** Pistillate flower (gynoecium) with vestigial abaxial glandular appendage. **(E)** Pistillate flower with well-developed abaxial glandular appendage. **(F–I)** Successive developmental stages of pistillate flowers lacking glandular appendage. Scale bars = 30 μm in **(A–I)**.

Single-flowered pistillate reproductive units develop in shoot nodes between the radii of the leafy segments (Figure 8A), whereas the vegetative branches are axillary (not shown). The growth of the vegetative segments is initially slow, so that they are shorter than the gynoecium at the stage when a central depression becomes visible in the latter (Figure 3B) and subsequent stages (Figures 3C,F).

**FIGURE 9:**
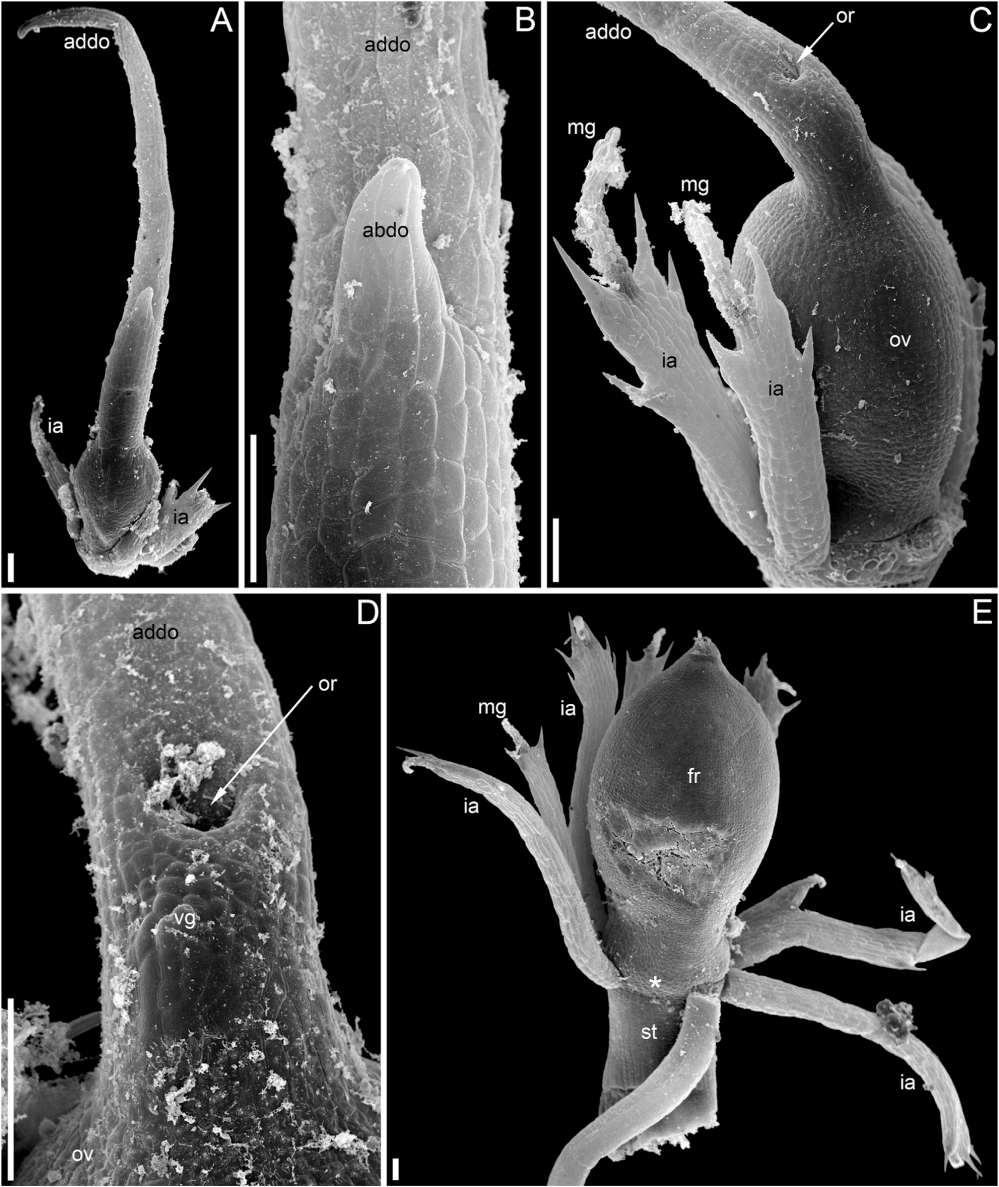
Pistillate flower and fruit morphology of *Ceratophyllum tanaiticum* (SEM). **(A)** Abaxial view of flower with long adaxial and short abaxial distal gynoecial outgrowth. **(B)** Detail of **(A)** showing abaxial outgrowth. The gynoecium orifice is hidden by the abaxial outgrowth. **(C)** Side view of flower with a single (adaxial) distal gynoecial outgrowth; two involucral appendages removed, but their scars are visible in the bottom-right part of the image. **(D)** Detail of abaxial view of gynoecium similar to that in **(C)**, but with a vestigial gland below the orifice. **(E)** Young fruit; note the ab initio absence of involucral appendages on one side of the fruit base (asterisk). Scale bars = 100 μm in **(A–E)**.

**FIGURE 10:**
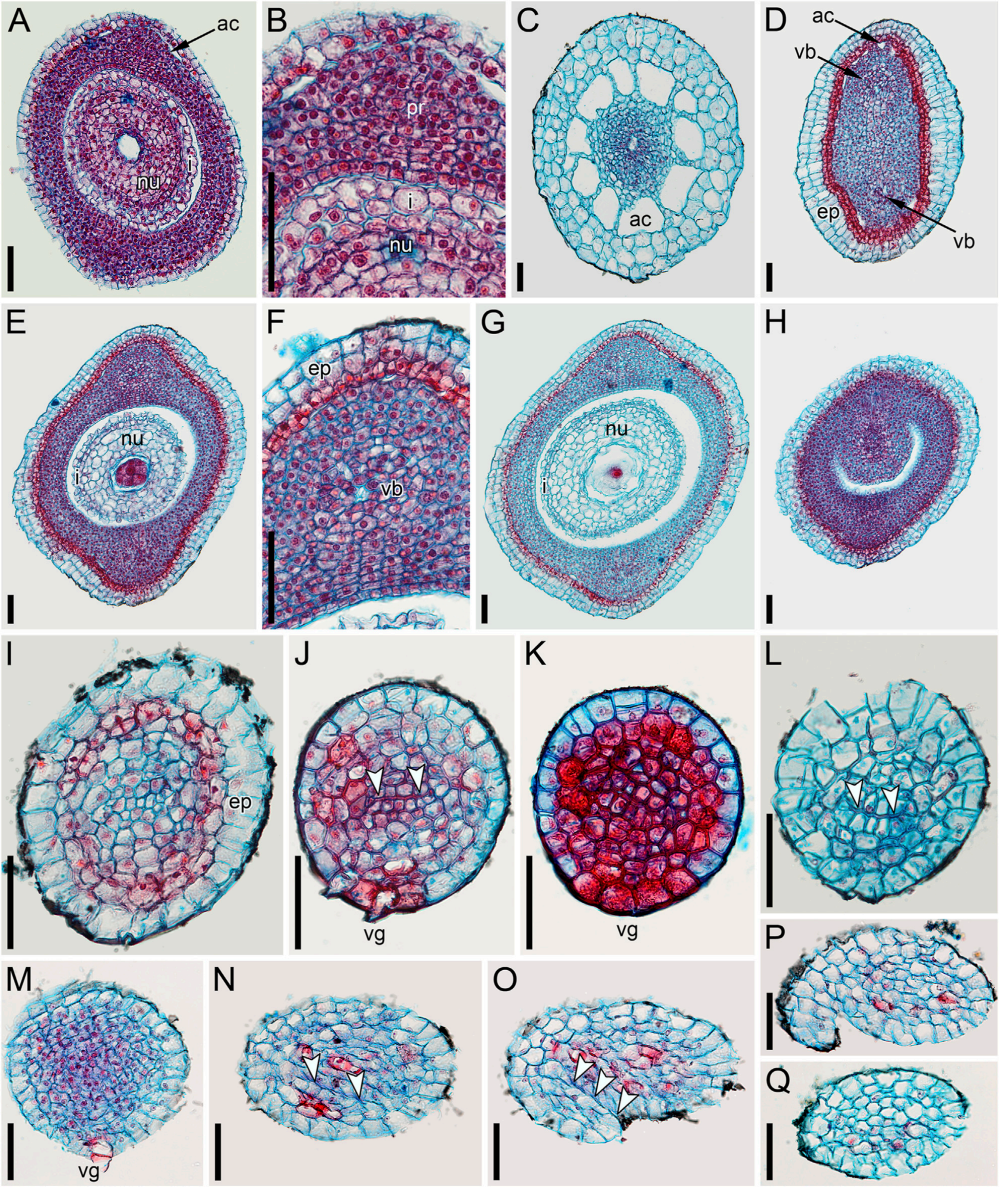
Pistillate flower anatomy of *Ceratophyllum tanaiticum* **(A–L)** and *C. submersum* **(M–Q)**, transverse sections, LM. Adaxial side up in all images. **(A,B)** Midsection of anthetic ovary **(A)** and detail of its adaxial side showing poorly recognizable procambium strand **(B)**. **(C–L)** Ascending serial sections of postanthetic flower similar to that in Figure 9E; involucral appendages removed before sectioning. **(C)** Stalk of pistillate reproductive unit. **(D)** Below the ovary locule and above the insertion of involucral appendages. **(E–H)** Ovary locule. Note that note that the *epidermis* is biseriate in **(D–H)**. **(E)** Bottom of the ovule showing globular proembryo (red cells in the centre). **(F)** Detail of adaxial part of the ovary wall from **(E)** showing vascular bundle. **(G)** Middle part of the ovule with a large endosperm cell in the centre. **(H)** Level of ovule attachment. **(I–L)** Above the ovary locule (arrowheads, stylar canal). **(J,K)** Level of vestigial gland. **(M–Q)** Ascending serial sections of anthetic flower above the ovary locule (arrowheads, stylar canal). The flower is similar to that in Figures 11D, 12D. **(M)** Level of vestigial gland (only two gland cells visible). **(N)** Above the gland. **(O)** Asymmetric gynoecium orifice. **(P)** Asymmetric proximal part of adaxial distal outgrowth of gynoecium. **(Q)** Symmetrical distal part of the outgrowth. Scale bars = 50 μm in **(A–Q)**.

Anthetic pistillate reproductive units are almost sessile, but postanthetic units (young fruits) are stalked. The stalk is situated below the whorl of the involucral appendages (Figures 1D, 9E). The stalk is circular in cross-section and has a ring of air canals (Figure 10C). The appendages are inserted in a whorl along the entire circumference around the gynoecium or they are absent on one side (Figure 9E). Each appendage has a distal mucilaginous gland and acute unicellular teeth on either side below the gland; additional teeth can also occur along the length of the appendage (Figures 9C,E). At the youngest available stages, the involucre of the pistillate flower forms a slightly lobed belt at the base of the gynoecium (Figure 8B).

The adaxial and abaxial sides of the rim around the gynoecium orifice are equal at the earliest available stages (Figures 3B,C). Most flowers then develop a long and entire cylindrical outgrowth on the adaxial side (Figures 3F–H–H, 9C,D), with its base incurved towards the shoot apex. In one flower, we observed a clearly defined triangular abaxial outgrowth much shorter than the adaxial outgrowth (Figures 9A,B). Another flower, studied at an early developmental stage before expansion of the adaxial outgrowth, possessed a well-developed and conspicuous glandular abaxial appendage (Figure 8E). Other flowers possessed a vestigial gland on the abaxial side at the level between the ovary and the gynoecium orifice (Figures 9D, 10J,K).

Anthetic (Figures 10A,B) and postanthetic (Figures 10D–L) gynoecia are anatomically similar with the difference that the latter show recognizable vascular bundles rather than scarcely visible procambium strands, biseriate epidermis up to the level of the ovule attachment and developing endosperm and embryo. The ovary is unilocular. The gynoecium is elliptical in cross-section below the ovary locule (Figure 10D), with two vascular bundles, one adaxial and the other abaxial. Outside each bundle, there are narrow peripheral air canals (Figures 10A,B,D) that are not directly continuous with those of the flower stalk (Figure 10C). The ovary has a single pendent ovule (Figures 10A,E–H) attached adaxially, with micropyle facing the base of the ovary. The adaxial bundle supplies the ovule and extends up to the level of ovule attachment. Neither bundle is discernible above the ovule attachment. The stylar canal is postgenitally closed in sections above the ovule, where a distinct line of closure is present (Figures 10I–L). A vestigial gland on the abaxial side of the gynoecium above the ovary is anatomically conspicuous (Figures 10J,K).

### Pistillate Flower Structure and Development in *Ceratophyllum submersum*

**FIGURE 11:**
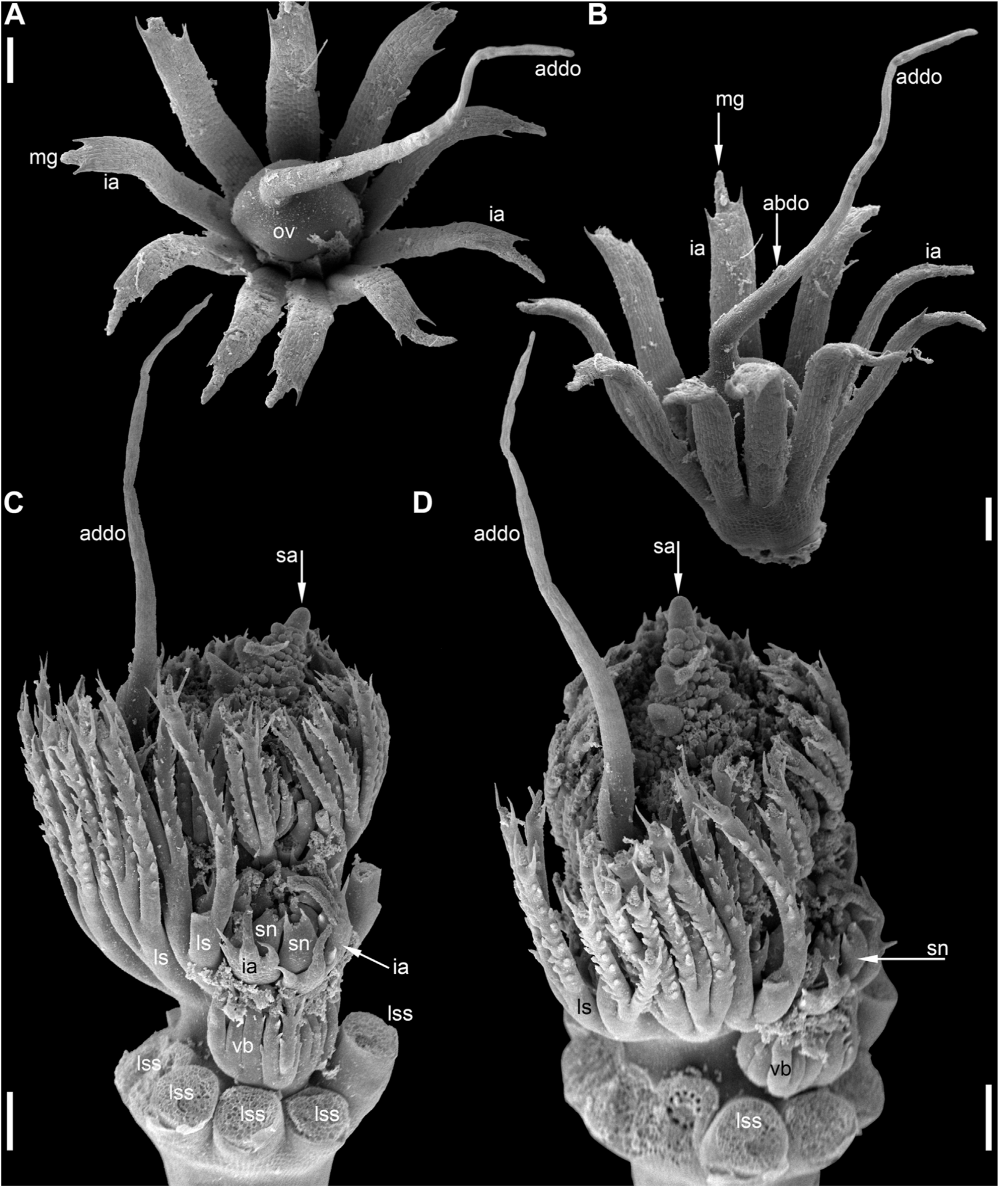
Reproductive structures of *Ceratophyllum submersum* (SEM). **(A,B)** Two views of pistillate reproductive unit. **(C,D)** Two views of shoot apex with anthetic pistillate reproductive unit and pre-anthetic staminate reproductive unit located a node below. There is also a vegetative branch located right below the staminate reproductive unit in yet another node. The staminate reproductive unit has numerous stamens and a peripheral whorl of involucral appendages. Scale bars = 300 μm in **(A–D)**.

**FIGURE 12:**
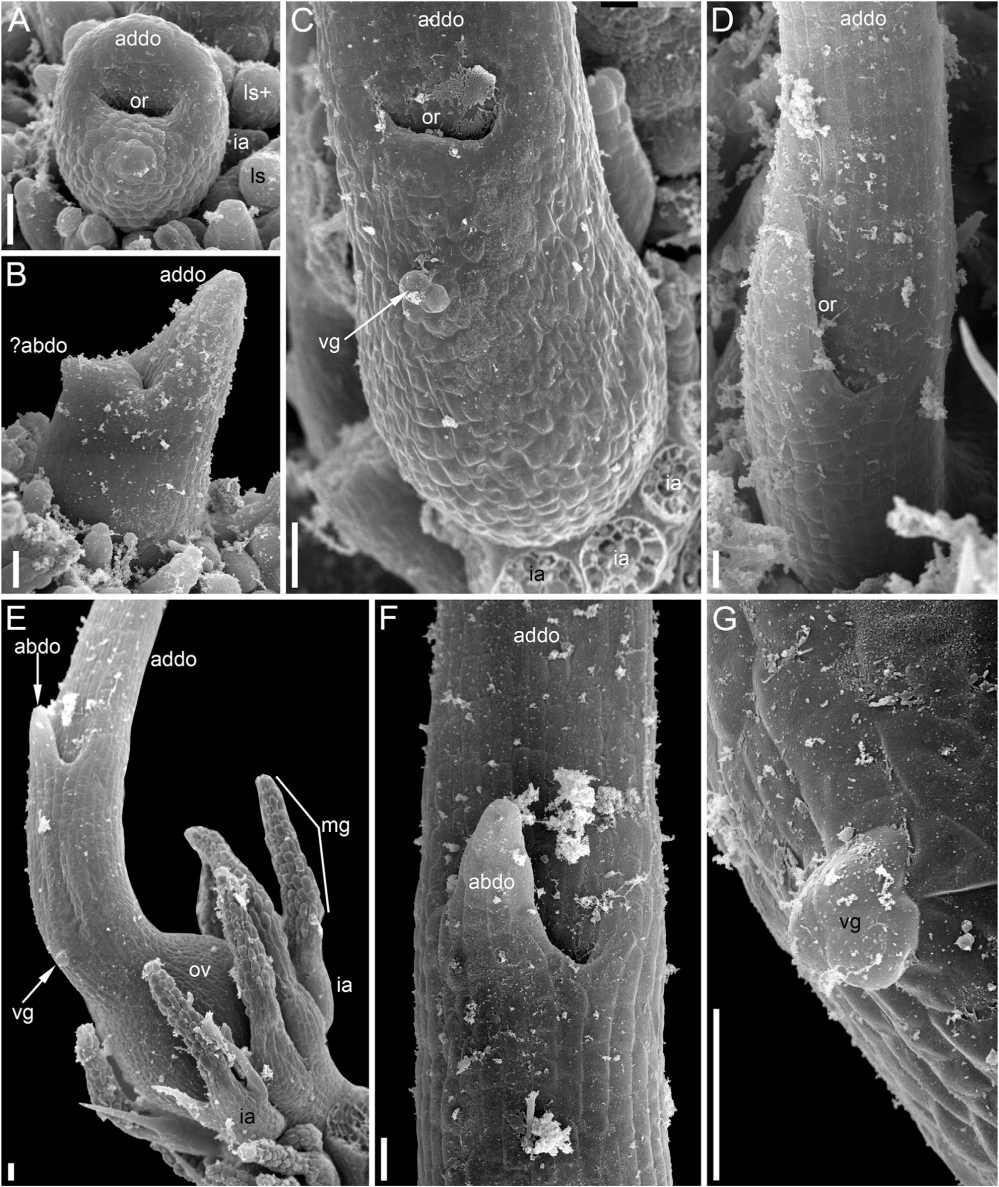
Pistillate reproductive units of *Ceratophyllum submersum* (SEM). **(A–C)** Successive developmental stages. **(A,C)** Adaxial side up; **(B)** adaxial side right. Each reproductive unit has a pistillate flower surrounded by a whorl of involucral appendages. The involucral appendages are removed in **(C)**, but their scars are visible. In **(A,B)**, the involucral appendages and leafy segments of stem nodes are yet small and densely crowded, so that it is difficult to distinguish them from each other without further dissection in **(B)**. **(D)** Detail of anthetic gynoecium seen from the abaxial side; the gynoecial orifice is asymmetrically spaced. **(E–G)** Anthetic gynoecium with two distal outgrowths of unequal size. **(E)** General view from lateral side. **(F)** View from abaxial side; the gynoecial orifice is behind the short abaxial outgrowth. **(G)** Detail of **(E)** with vestigial gland. Scale bars = 300 μm in **(A–G)**.

As in the other two species, the single-flowered pistillate reproductive units develop in shoot nodes between the radii of the leafy segments, whereas the vegetative branches are axillary (Figures 11C,D). Growth of the vegetative segments is much delayed relative to pistillate flowers. At the stage when an apical depression in the gynoecium is clearly visible, the vegetative appendages of the same node are much shorter than the gynoecium and only slightly longer than the involucral appendages of the flower (Figure 12A).

The pistillate flower is very shortly stipitate and surrounded by a whorl of about ten involucral appendages of equal size (Figures 11A,B). In very young gynoecia, the abaxial and adaxial margins of the orifice are almost equally developed (Figure 12A), but a long distal outgrowth develops rapidly on the adaxial (or possibly adaxial-transversal) side (Figures 11A,B, 12B–D–D). At anthesis, the adaxial outgrowth is longer than all adjacent vegetative segments and well exceeds the shoot apex (Figures 11C,D). Development varies on the abaxial side of the gynoecium. A short tooth-like distal outgrowth and/or a glandular (apparently mucilaginous) appendage may develop here (Figures 10M, 12B,C, E–G–G). The non-glandular outgrowth, when present, remains at the level of the gynoecial orifice. Its position is not median-adaxial but shifted laterally (Figures 12E,F). In some gynoecia, the short outgrowth appears to be unilaterally united with the long outgrowth (Figures 10M–QM–Q Figures 10M–Q, 11D, 12D). The glandular abaxial appendage is very short, sometimes represented by a few cells and can be interpreted as vestigial gland (Figures 10M, 12G). In younger gynoecia, it is located closer to the gynoecial orifice (Figure 12C). At older stages with elongation of the style-like part of the gynoecium, the vestigial gland is located much below the orifice, but always above the level of the ovary (Figure 12E). Entire gynoecia observed at various developmental stages are characteristically incurved towards the shoot apex (Figures 11C,D). The ovary has a single pendent ovule attached adaxially (not shown).

### Evolution of Syncarpy

**FIGURE 13:**
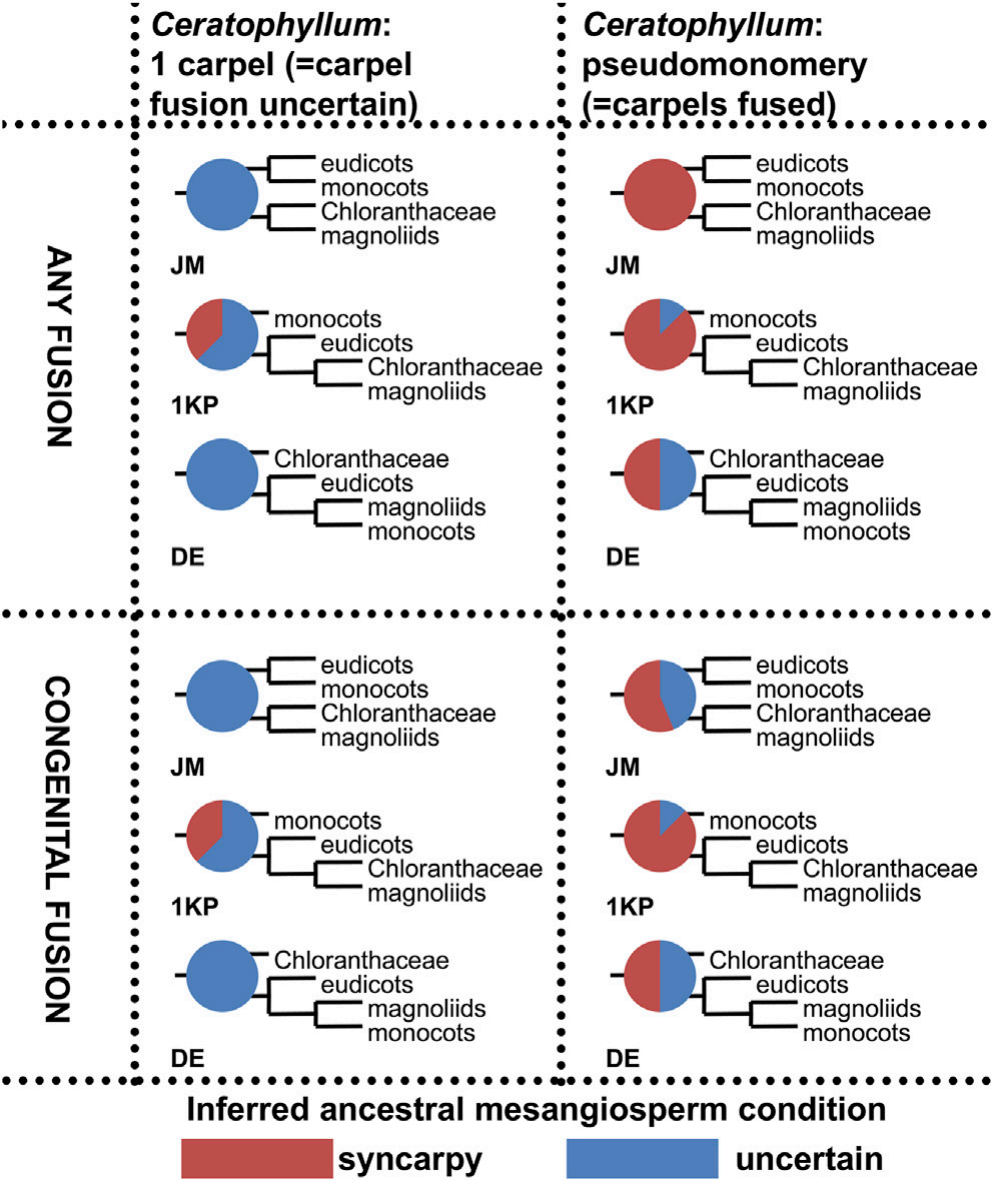
Summary of ancestral state reconstructions with parsimony of fusion between carpels in mesangiosperms on different tree topologies and with two types of coding for the state ‘syncarpy’ (any fusion = carpel fusion may be congenital or postgenital; congenital fusion = carpel fusion concerns only congenital fusion) in angiosperms. For more detail, see Supplementary File 2. Each pie chart shows the ratio between inferred ancestral mesangiosperm conditions among 32 tree topologies that differ in placement of *Ceratophyllum*, *Nuphar*, *Euptelea* and Tofieldiaceae. Each diagram shows what pattern of relationships between monocots, eudicots, magnoliids and Chloranthaceae was fixed in each set of 32 tree topologies.

Ancestral state reconstruction of carpel fusion (apocarpy vs. syncarpy) with parsimony was performed on three sets of 32 phylogenetic trees, each with the relative positions of monocots, eudicots, Chloranthaceae and magnoliids fixed as in the JM, 1 KP or DE topologies (Figure 2A). In each set of trees, we considered all possible combinations of positions of *Ceratophyllum*, *Euptelea*, Tofieldiaceae and *Nuphar* (Figure 2B). In total, 3*32 = 96 tree topologies were analysed (Table 1, Figure 13, Supplementary File 2).

1) To what extent does the phylogenetic context help in disentangling the gynoecium morphology of *Ceratophyllum* as pseudomonomerous or truly monomerous? Table 1 presents parsimony reconstructions of the ancestral gynoecium in a stem-group lineage of *Ceratophyllum* under different phylogenetic topologies, showing the extent to which phylogenetic placement aids homology reconstruction. The gynoecium morphology of *Ceratophyllum* is scored as unknown in the analyses summarized in Table 1. With *Ceratophyllum* scored as unknown, we inferred the gynoecium condition for its stem-group lineage as either uncertain or syncarpous (Table 1). From the 96 tree topologies summarized in Table 1, only eight were sensitive to the way in which the character of gynoecium fusion was coded, either when syncarpy included both congenital and postgenital types of fusion (i.e., original coding from Sauquet et al., 2017), or when syncarpy included congenital fusion only. Twenty topologies revealed syncarpy as the ancestral condition for *Ceratophyllum,* with either type of coding for syncarpy. In the remaining 68 topologies, the ancestral condition was ambivalent. When *Euptelea* is sister to all other Ranunculales and/or *Ceratophyllum* is sister to all other mesangiosperms, the gynoecium condition of *Ceratophyllum* was inferred as uncertain irrespective of all other parameters (Table 1).
2) To what extent are reconstructions of gynoecium evolution in angiosperms sensitive to contrasting interpretations of the pistillate flower in *Ceratophyllum*?
  2a) In the angiosperm-wide data set, when no difference was made between congenital and postgenital fusion for the character state ‘syncarpy’ (i.e., presence of carpel fusion) the ancestral condition inferred for mesangiosperms was strongly sensitive to the scoring of *Ceratophyllum* in the set of trees with JM topologies (Figure 13, two uppermost diagrams). When carpel fusion of *Ceratophyllum* was scored as uncertain (the original scoring of Sauquet et al., 2017), the occurrence of free or united carpels in the ancestor of mesangiosperms was equally parsimonious irrespective of the positions of *Ceratophyllum*, *Euptelea*, Tofieldiaceae and *Nuphar* in the JM tree. In contrast, when *Ceratophyllum* was scored as having fused carpels, syncarpy was consistently inferred as the ancestral condition of mesangiosperms in JM trees. In other words, rescoring only one terminal group in such a large data set considerably changed the inferences for ancestral states in JM trees. In 1 KP and DE trees, the effect of rescoring was less strong, but still present (Figure 13).
  2b) In the angiosperm-wide data set, when syncarpy was restricted to congenital fusion only, some topologies were sensitive to the scoring of *Ceratophyllum*, but other topologies resulted in an uncertain condition for the ancestor of mesangiosperms irrespective of interpretation of *Ceratophyllum* (Figure 13).

## Discussion

### Problematic Organ Homologies in Pistillate Reproductive Structures of *Ceratophyllum*: Historical Background

**FIGURE 14:**
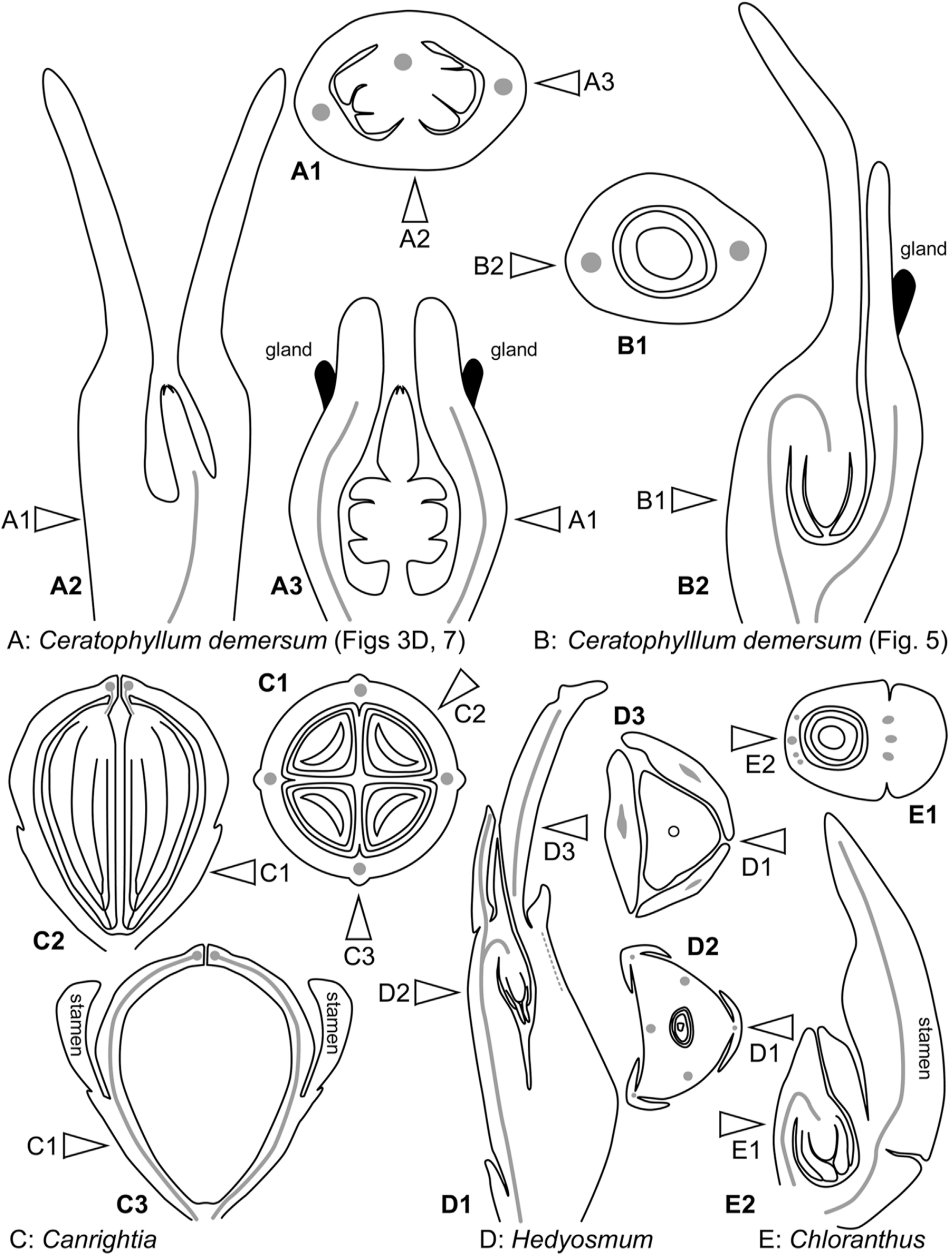
Diagrams of pistillate or bisexual flower morphology in *Ceratophyllum* and some of its possible phylogenetic relatives. In each case, longitudinal and cross-sections are provided, with their positions reciprocally indicated by arrows. **(A)** One of the rare conditions observed here in *Ceratophyllum demersum* (Figures 3D, 7). **(B)** The most common type of pistillate flower in *Ceratophyllum*. **(C)** Cretaceous fossil *Canrightia* (modified from Friis and Pedersen, 2011). **(D,E)** Extant members of Chloranthaceae. **(D)** *Hedyosmum* (modified from Endress, 1971). **(E)** *Chloranthus* (modified from Endress, 1987b).

Even though the pistillate flowers of *Ceratophyllum* (not considering the involucre) are rather simple structures, their morphological interpretation is far from straightforward. The main problem is a long filiform distal extension to the gynoecium (Figure 14B), sometimes called the stigma, which is normally attached on the same side as the ovule (Troll, 1933; Shamrov, 1983; Endress, 1994; Igersheim and Endress, 1998; Endress, 2001; Iwamoto et al., 2003; Endress and Doyle, 2015). This extension can be interpreted as median-adaxial in its attachment relative to the main axis on which the flowers develop (but note that flower-subtending bracts are lacking in *Ceratophyllum* unless all leafy segments in each node are interpreted as parts of the same dissected leaf with a group of collateral axillary buds: Raynal-Roques, 1981; Rutishauser and Sattler, 1987; Rutishauser, 1999; Iwamoto et al., 2015). Most commonly, distal carpel extensions are dorsal in basal angiosperms (i.e., angiosperms other than monocots and eudicots). Non-receptive dorsal outgrowths that function as osmophores are present on the carpels of *Nymphaea* and related genera (Endress, 2001; Zini et al., 2019). In other taxa possessing a distal carpel extension (e.g., the magnoliid family Lauraceae), an extension producing a plicate style/stigma is also located in the dorsal part of the carpel (Endress, 2015). If the flower of *Ceratophyllum* has only a single carpel which is ascidiate, as widely interpreted, precise identification of its ventral and dorsal side is problematic. However, the ventral side of the carpel is in an adaxial position in most angiosperms with unicarpellate flowers, suggesting that the *Ceratophyllum* outgrowth could be ventral rather than dorsal. Whatever interpretation is followed, the relative placement of the ovule with respect to the distal extension is uncommon for angiosperms (Igersheim and Endress, 1998). The ovule is ventral in most uniovulate angiosperm carpels, and if the ovule is ventral in *Ceratophyllum*, then the appendage is indeed ventral. In summary, the occurrence of a distal outgrowth on the same side as the ovule found in *Ceratophyllum* is uncommon for uniovulate angiosperm carpels (Igersheim and Endress, 1998).

A possible explanation for the unusual relative positions of the ovule and the distal extension is that the gynoecium of *Ceratophyllum* is pseudomonomerous, consisting of two carpels with the sterile carpel bearing the long distal extension (de Klercker, 1885). This hypothesis was rejected by Endress (1994) based on the lack of evidence for two carpels at early developmental stages, but it was supported by Shamrov (2009) based on observations of variation in gynoecium anatomy. Iwamoto et al. (2003) reported a small mucilaginous appendage in the gynoecium of *Ceratophyllum demersum*, similar to the mucilaginous glands that develop at the tips of leaf segments and stamens in this species. The appendage is located in an abaxial position and above the ovary. It rapidly abscises and its position can no longer be traced in anthetic flowers using light microscopy, perhaps explaining why this structure was overlooked by most earlier authors, though de Klercker (1885) nicely illustrated longitudinal sections of *C. demersum* at different developmental stages. A gland similar to that of *C. demersum* was found by Strasburger (1902) in *C. submersum.* Based on the occurrence of similar glands at the tips of the vegetative leaves, involucral segments and stamens, Iwamoto et al. (2003) suggested that the mucilaginous gynoecial appendage could indicate a carpel tip, and the presence of only one appendage indicates a single carpel. Note that under the interpretation of Iwamoto et al. (2003), the long adaxial outgrowth of the gynoecium does not represent a carpel tip.

### Glandular Appendages of the Gynoecium

Our study has confirmed the occurrence of the glandular (mucilaginous) appendage in the abaxial position in gynoecia of *C. demersum* (Figure 14B) and *C. submersum*, as described by Iwamoto et al. (2003) and Strasburger (1902). We found variation in the presence or absence of an externally visible glandular appendage in both *C. submersum* and *C. tanaiticum*. In flowers of *C. submersum*, the gland (when present) is very small and apparently rudimentary. Our anatomical data has revealed what can be interpreted as a vestigial gland in exactly the same position, even in gynoecia of *C. submersum* and *C. tanaiticum* lacking an externally recognizable gland.

Our data do not support the proposal of Iwamoto et al. (2003) that the mucilaginous glandular appendage of the gynoecium indicates the carpel apex. In our view, its position well below the orifice of the fully developed gynoecium makes this interpretation highly problematic, as it requires interpretation of the long tubular part of the gynoecium (“style”) above the appendage as formed by a secondary carpel margin. Furthermore, our observations of gynoecia with two glandular appendages in *C. demersum* also contradict this theory.

We propose an alternative hypothesis, that the ephemeral glandular appendage represents the tip of a rudimentary perianth member or staminode, thus highlighting the inferior nature of the ovary in *Ceratophyllum*. As outlined in the next paragraph, this hypothesis fits well with a potential relationship between *Ceratophyllum* and Chloranthaceae. Three other possible phylogenetic positions of *Ceratophyllum* (Figure 2B) do not imply its sister relationship to a clade with an ancestrally inferior ovary, though epigynous flowers have evolved independently in many lineages within monocots, eudicots and magnoliids.

The family Chloranthaceae has received special attention as the potential sister group of *Ceratophyllum* because of several shared morphological characters (Endress and Doyle, 2009; Endress and Doyle, 2015; Doyle and Endress, 2018). Among the four extant genera of Chloranthaceae (*Ascarina, Chloranthus, Hedyosmum, Sarcandra*), the ovary is inferior in pistillate flowers of *Hedyosmum*, where there are three perianth organs (Endress, 1971; Endress, 1987b; Doyle and Endress, 2014; Sokoloff et al., 2018b). In *Ascarina*, the gynoecium is the only organ of the pistillate flower, so the condition of ovary position is unknown relative to other organs (Endress, 1987b; Doyle and Endress, 2014). In the two extant genera of Chloranthaceae with bisexual flowers, there is either a stamen (*Sarcandra*) or tripartite androecium (*Chloranthus*) attached abaxially to the ovary (Swamy, 1953; Endress, 1987b), i.e. in a position that is similar to that of the glandular appendage of *Ceratophyllum*. Doyle and Endress (2014) avoided scoring the ovary of *Sarcandra* and *Chloranthus* as inferior (or semi-inferior), but this is a question of terminology rather than interpretation; the inferred similarity with *Ceratophyllum* is more important. A semi-inferior ovary is also found in fossils interpreted as most likely related to extant Chloranthaceae and *Ceratophyllum*, including *Canrightia* (one species, Early Barremian to Early Albian of Portugal, Friis and Pedersen, 2011) and *Zlatkocarpus* (two species, Cenomanian of Czech Republic, Kvaček and Friis, 2010) as well as in an extinct genus of Chloranthaceae, *Canrightiopsis* (three species, Albian of Portugal Friis et al., 2015). The morphological phylogenetic analysis of Kvaček et al. (2016) placed *Canrightia* and *Zlatkocarpus* as two successive sister groups of a clade that includes extant Chloranthaceae as well as *Ceratophyllum*, with the inferior ovary as an ancestral condition. Remarkably, the potential perianth (‘floral cup’) of *Zlatkocarpus* is much more pronounced on the abaxial side of the flower (Kvaček and Friis, 2010).

The ovary of *Ceratophyllum* was previously interpreted as superior, apparently because of the widespread view that the sterile involucre around the gynoecium is a perianth. Assuming that this involucre does not belong to the pistillate flower (Les, 1986; Endress, 1994; Endress, 2001; Endress and Doyle, 2009; Endress and Doyle, 2015), our interpretation of the ovary as inferior is plausible. The sporadic occurrence of two mucilaginous appendages in *C. demersum* also fits with this interpretation.

### Plasticity in the Number of Distal Outgrowths

Our study has revealed variation in the number of distal outgrowths in all three species examined. Reports of two sporadic distal outgrowths (or a “bifid style”) in *Ceratophyllum* are not new (Arber, 1920; Les, 1986). The range of variation found within *C. demersum* could indicate either 1) a type of fasciation resulting in a split of the distal outgrowth, or 2) the evolutionary origin of the typical single median adaxial outgrowth by fusion of two transversal-adaxial parts, belonging either to the same carpel (2a) or to different carpels (2b).

The presence of two equal stigmatic appendages in a transversal position is known in carpels of some other angiosperms. Among members of the ANA grade, this condition is pronounced in *Austrobaileya* (Igersheim and Endress, 1998). It is also known in some magnoliids (in Annonaceae and Aristolochiaceae: Igersheim and Endress, 1998). A laterally two-lobed carpel apex is present in some monocots, most remarkably in the aquatic family Hydrocharitaceae (reviewed by Igersheim et al., 2001). Importantly, these examples are all from multicarpellate gynoecia, so the identity of the two stigmatic lobes cannot be questioned. Features that are found in *Ceratophyllum* and not in other angiosperms with two lateral stigmatic appendages per carpel include the often unequal length of the appendages, their frequent unilateral fusion and their variable position (an almost abaxial appendage is recorded in *C. submersum* and *C. tanaiticum*). Therefore, we consider it unlikely that the two lobes belong to the same carpel (hypothesis 2a, above).

Distinguishing between the fasciation hypothesis (1) and the two-carpel hypothesis (2b) is more problematic. Placing the observed variation in a phylogenetic context is especially important to resolve such problems, but in the case of *Ceratophyllum* it provides little (if any) clarification (Table 1). Our discovery of a proximally bilocular and distally unilocular gynoecium with two equal distal outgrowths (Figures 3D, 7, 14A) could provide evidence in favour of the ancestral presence of at least two carpels in *Ceratophyllum*. We are inclined to reject the fasciation hypothesis (1) because the proximally bilocular and distally unilocular gynoecium (Figures 3D, 7, 14A) does not represent a ‘simple’ duplication of what is most commonly present in gynoecia of *Ceratophyllum*. We prefer the two-carpel hypothesis (2b) and interpret the ovary of this gynoecium as having a proximal bilocular and distal unilocular zone. Each locule of the biolocular zone has an ovule attached to the septum (axile placentation), and the third ovule is attached at the very base of the unilocular zone in a parietal placenta (Figure 14A). The two locules are in the median plane of the flower, while the two distal outgrowths are in the transverse plane. With respect to their position, these outgrowths resemble the commissural stigmas found, for example, in Papaveraceae (Ranunculales, basal eudicots) and some Aristolochiaceae (Piperales, magnoliids) (Igersheim and Endress, 1998; Endress and Igersheim, 1999). Commissural stigmas of syncarpous gynoecia are located in radii alternating with the dorsal radii of the carpels. They could be viewed as congenitally united distal lobes of adjacent carpels.

Interpretation of the gynoecium of *Ceratophyllum* as reduced from a non-monomerous syncarpous condition with commissural stigmas provides the simplest explanation of gynoecium variation in *C. demersum*. As outlined above, the main problem with a unicarpellate interpretation of the most common gynoecium type in *Ceratophyllum* is that the long distal appendage is positioned in the same radius as the ovule. Such an arrangement could readily be envisaged in a pseudomonomerous gynoecium. Progressive reduction of the adaxial carpel in a gynoecium similar to that in Figures 3D, 7 should result in progressive unilateral fusion of the two distal appendages on the adaxial side of the flower. Based on these ideas, it is difficult to determine whether the single ovule normally found in *Ceratophyllum* belongs to the symplicate or synascidiate zone. Moreover, recognizing these zones in such reduced pseudomonomerous gynoecia is technically impossible (see Bachelier and Endress, 2007). Shamrov (2009) illustrated a gynoecium with two ovules on either side of a unilocular (thus most likely symplicate) ovary, but he provided no information on the position of the distal appendage(s). It is even possible that the ancestral gynoecium of *Ceratophyllum* had more than two carpels. Evidence for this is the nearly abaxial position of the smaller distal outgrowth found in *C. tanaiticum*.

The gynoecium morphology of *Ceratophyllum* should be viewed in the context of morphological idiosyncrasies of submerged water plants and the possible functional load of observed characters. The long distal gynoecial outgrowth of *Ceratophyllum* resembles the narrow filiform organs commonly found in many submerged aquatics. However, on closer inspection the distal gynoecial outgrowth of *Ceratophyllum* is not closely similar to its leafy segments or involucral appendages, because the latter are pronouncedly bifacial organs with marginal teeth. The distal outgrowth(s) must play a functional role during pollination, because in living plants it is positioned along the water surface where pollen grains are released from floating anthers (e.g., Strasburger, 1902; Raynal-Roques, 1981; Shamrov, 1983). Functional interpretations may be problematic when there are two distal outgrowths, one long and filiform and the other very short (Figures 9A,B, 12E,F). The sporadic presence of the second, short outgrowth apparently cannot be explained by hydrodynamic reasons or pollination biology. More likely, its presence is related to general patterns of morphological variation in the *Ceratophyllum* flower.

### Non-Monomerous Versus Monomerous Gynoecium Evolution in *Ceratophyllum* and Extant Chloranthaceae

According to the most widely accepted view, an important character shared by *Ceratophyllum*, Chloranthaceae and most members of the basal angiosperm grade is the presence of ascidiate carpels lacking a plicate zone, so that this condition is probably plesiomorphic in angiosperms (Endress and Igersheim, 2000; Endress, 2001; Endress, 2015). Flowers of *Ceratophyllum* and Chloranthaceae are usually viewed as unicarpellate (i.e., with a truly monomerous gynoecium). However, using the hypothesis of a non-monomerous gynoecium in *Ceratophyllum* and the strong morphological evidence for its potentially close affinities with Chloranthaceae (Endress and Doyle, 2009; Endress and Doyle, 2015), it is useful to review earlier discussion of potential pseudomonomery in Chloranthaceae (Edwards, 1920; Swamy, 1953; Endress, 1971; Endress, 1987b; Endress, 2001; Sokoloff, 2015).

All extant Chloranthaceae possess a gynoecium with a congenitally continuous wall and a single pendent ovule (Figures 14D,E). Such a gynoecium fits a single ascidiate carpel (Endress, 1971; 1987b; Endress, 2001), despite the remarkable vascular supply of the single ovule of *Hedyosmum*. A complete and detailed description of floral vasculature in *Hedyosmum* was provided by Endress (1971) for *H. mexicanum.* The wall of the inferior ovary has three major longitudinal bundles, one in a median adaxial and two in transversal-abaxial positions. These bundles split distally to form an outer branch serving a tepal and an inner branch that forms a circular portion of vascular tissue just below the gynoecium orifice. The stylar strand joins this circular vascular connection on the abaxial side. The bundle that serves the ovule is derived from the inner branch of the adaxial major longitudinal bundle of the ovary wall, immediately below (almost at the level of) the circular vascular connection (Endress, 1971). Occasionally, one of the two transversal-adaxial major longitudinal bundles lacks an inner branch and the vascular region below the gynoecium orifice is thus one-sided rather than circular (Endress, 1971). The drawing of Swamy (1953—his figure 20) showing direct ovule innervation in *H. nutans* by three bundles, was apparently modified from an earlier drawing of *H. nutans* (Edwards, 1920), with the difference that the latter illustrated a stylar bundle that was omitted by Swamy. Edwards (1920) stated that the stylar strand is connected with one or possibly with all three ovular bundles and showed the stylar bundle as ending blindly (apparently the reason why the bundle was subsequently omitted by Swamy). It is therefore likely that a distal circular vascular region is present in *H. nutans* in the same way as described in detail by Endress (1971) in *H. mexicanum*, a pattern of ovule innervation that is similar to that in fossil *Canrightia*.

### *Canrightia*: an Early Cretaceous Fossil with Enigmatic Gynoecium Morphology Potentially Related to Chloranthaceae and *Ceratophyllum*

The Early Cretaceous fossil *Canrightia resinifera* (Friis and Pedersen, 2011), whose close relationship with both Chloranthaceae and *Ceratophyllum* is rather plausibly proposed (Kvaček et al., 2016; Doyle and Endress, 2018) has a noteworthy gynoecium that could be interpreted as syncarpous (parasyncarpous: Friis and Pedersen, 2011; Doyle and Endress, 2014; Doyle and Endress, 2018). It has a semi-inferior unilocular ovary with two to five symmetrically arranged pendent ovules (Figure 14C). The symmetrically spaced axial vascular bundles of the ovary wall unite with each other distally at the level of ovule attachment (Friis and Pedersen, 2011). This vascular pattern resembles that observed in extant *Hedyosmum*, except that the ovule of *Hedyosmum* is located in the radius of the adaxial vascular bundle. Doyle and Endress (2018) preferred an evolutionary scenario in which the common ancestor of *Canrightia* and a clade that includes extant Chloranthaceae and *Ceratophyllum* possessed three free carpels. However, this scenario implies that fusion between putatively ascidiate carpels of the hypothetical ancestor resulted in a unilocular rather than plurilocular gynoecium in *Canrightia*. A pseudomonomerous interpretation for *Ceratophyllum* prompts an alternative scenario, in which the common ancestor of the clade that includes Chloranthaceae and *Ceratophyllum* possessed a syncarpous gynoecium similar to that of *Canrightia* (Friis et al., 2015; Doyle and Endress, 2018). An intriguing point is that the carpels of *Canrightia* (if the gynoecium is viewed as syncarpous) are entirely plicate with no trace of a synascidiate zone; the gynoecium appears to be entirely symplicate. If this condition is accepted as ancestral for the group, then the gynoecium of extant Chloranthaceae only mimics a solitary ascidiate carpel and the resemblance between their gynoecium and the individual ascidiate carpels of taxa such as *Amborella* (the putative sister taxon to all other extant angiosperms) would be an impressive example of evolutionary convergence.

Interpreting the gynoecium of *Canrightia* as syncarpous is based solely on the obvious fact that no other extant or extinct angiosperm has a multicarpellate gynoecium in which the individual carpels are constructed like the entire gynoecium of *Canrightia*, which is too symmetrical to be a single ascidiate carpel (including the ring-like attachment of the pendent ovules). The origin and homologies of the angiosperm carpel remain largely unknown (e.g., Doyle, 2008; Doyle, 2012; Sokoloff et al., 2017). Alternatively, it could be argued that *Canrightia* shows a rather plesiomorphic type of ascidiate carpel. Similar triradiate (rather than bilateral) symmetry of the vasculature is known in the ascidiate carpel of two water-lily families, Cabombaceae (Moseley et al., 1984; Endress, 2005) and Hydatellaceae (Sokoloff et al., 2013b).

### *Ceratophyllum* and Its Potential Early Cretaceous Relatives Possessing Uniovulate Ovaries

*Canrightia* represents the closest potential extinct relative of *Ceratophyllum* (and Chloranthaceae) that possesses more than one ovule per ovary. Other potential fossil relatives of *Ceratophyllum* and Chloranthaceae have an unilocular ovary (or fruit) with a single pendent orthotropous ovule (or seed). They should be scored as uncertain with respect to the character ‘fusion between carpels’ and therefore, in contrast with *Canrightia*, shed little light on the evolution of syncarpy. For completeness, the most important of other uniovulate fossils are discussed here (see also Supplementary File 3).

*Montsechia vidalii* from the Barremian of Spain (Gomez et al., 2015; Gomez et al., 2020) and *Pseudoasterophyllites cretaceus* from the Cenomanian of the Czech Republic (Kvaček et al., 2012, 2016) are preserved as compressions/impressions of branching shoots with numerous small entire leaves with opposite-decussate to spiral phyllotaxis. In *Montsechia*, the two types of phyllotaxis are restricted to long and compact shoot types, respectively. In *Pseudoasterophyllites*, the vegetative leaves and subtending leaves of the pistillate reproductive units (each unit producing a single fruit) are decussate, but the subtending bracts of staminate flowers (each possessing a single stamen) are spirally arranged. The two fossils both display leaf dimorphism with short and long leaves. Both fossils have long been considered problematic and several hypotheses exist on alternative affinities, including some potential non-angiosperm. However, recent phylogenetic analyses have placed *Pseudoasterophyllites* and *Montsechia* in a clade with *Ceratophyllum*, as sister to a clade comprising all extant Chloranthaceae plus their closest extinct relatives (Gomez et al., 2020; see also Kvaček et al., 2016; Doyle and Endress, 2018). *Montsechia* has been interpreted as the earliest known submerged aquatic angiosperm, if not the oldest macrofossil angiosperm (Gomez et al., 2015; Gomez et al., 2020). It has a thin cuticle and leaves with occasional stomata. The sparseness of the stomata could well represent an argument in favour of an aquatic habit in *Montsechia*, but their presence contradicts the idea of submerged growth; a more plausible possibility is that it was a floating plant (Doyle and Endress, 2018). Krassilov (2011) interpreted *Montsechia* as having relatively thick, succulent leaves and a heloxerophytic habit, growing in waterlogged periodically desiccated coastal marshes and sprouting leafy shoots with the rise of the lake level. Gomez et al. (2015), Gomez et al. (2020) did not address these ideas in detail, so their suggestion of a submerged aquatic habit for *Montsechia* requires further justification. An apparently succulent halophytic habit was also suggested for *Pseudoasterophyllites* (Kvaček et al., 2012; Kvaček et al., 2016). The absence of observed roots in both *Montsechia* and *Pseudoasterophyllites* (as well as stamen material in *Montsechia*) could reflect incomplete preservation.

A common feature of *Pseudoasterophyllites* and *Montsechia* is the absence of leaf stipules (Kvaček et al., 2016; Gomez et al., 2020), in contrast with extant Chloranthaceae, indicating strong variation in this diverse lineage. The pseudo-verticillate phyllotaxis of *Ceratophyllum* is plausibly interpreted as derived from decussate phyllotaxis with interpetiolar stipules, the stipules having been evolutionarily transformed into leaf-like organs (Iwamoto et al., 2015). Among angiosperms other than eudicots and Ceratophyllaceae, interpetiolar stipules are known only in Chloranthaceae. Thus, this feature could represent an important argument for a close phylogenetic relationship between Chloranthaceae and *Ceratophyllum*, as the ancestors of *Ceratophyllum* probably possessed decussate leaves with interpetiolar stipules. Interestingly, the occurrence of more than two stipules in an interpetiolar position is documented in *Ascarina lucida* (Jensen, 2021), resembling the unstable number of leaf-like structures in nodes of *Ceratophyllum*.

In *Pseudoasterophyllites*, the pistillate flowers each consist of a single gynoecium on a short stalk with one or two bracts, the entire unit borne in the axil of a leafy subtending bract (Kvaček et al., 2016). The gynoecium produces an elongated, somewhat curved, indehiscent fruit, with a single locule and a single pendent, orthotropous seed. There is a sessile stigma surrounding a near-apical short longitudinal slit. Although the surface of the fruit is slightly ribbed, vascular bundles are not preserved. In *Montsechia*, only the fruiting stage is known and there is no evidence for any floral elements except the gynoecium (Gomez et al., 2015; Gomez et al., 2020). The fruits usually develop in pairs at the apex of short shoots, with the smaller leaves forming a rosette. It is unclear whether two fruits develop from two different flowers or the same flower. The fruits are indehiscent and unilocular, each containing a single seed that develops from a pendent orthotropous and apparently unitegmic ovule. The unitegmic condition in *Montsechia* is an important potential synapomorphy with *Ceratophyllum*, though the condition is unknown in *Pseudoasterophyllites*.

In *Montsechia*, a small but conspicuous opening at the distalmost end of the fruit fossil could represent stigmatic tissue, comparable with the distal opening found in ascidiate carpels of Chloranthaceae and *Ceratophyllum* (Gomez et al., 2020). Alternatively, the opening found in the fossil could be the site of abscission of the distal part of the pistil, as in some extant species of *Ceratophyllum*, including *C. submersum* and *C. tanaiticum* (Kaden, 1953; present study: Figure 9E). In *Ceratophyllum*, the abscission zone is located below the gynoecium orifice (D.D. Sokoloff, E.S. El, unpubl. data). If the abscission hypothesis is taken into account, the number of stigmas should be considered uncertain in *Montsechia*. A vascular bundle in the pericarp of *Montsechia* is directed towards the seed hilum (Gomez et al., 2015; Gomez et al., 2020), but its position is not entirely clear. The reconstruction of Gomez et al. (2015: their Figures 3C,D) shows two fruits at the tip of a short shoot. Assuming that the two fruits are facing each other by their ventral sides, each fruit is illustrated as having a dorsal bundle and a dorsally attached seed. However, the photograph of a fruit pair in Gomez et al. (2020: their Figure 10A) is labelled as having a dorsal bundle in one fruit and an almost lateral bundle in the other fruit. As illustrated in Figure 10B of Gomez et al. (2020), the structure labelled as a vascular strand resembles one of the longitudinal folds of the pericarp (a similar ribbed fruit surface is found in *Pseudoasterophyllites,* Kvaček et al., 2016). In our view, such difficulties in interpretation make inferences on gynoecium morphology of *Ceratophyllum* highly problematic based on what is currently known in *Montsechia*.

Two species of fossil *Zlatkocarpus* from the Cenomanian of Czech Republic (Kvaček and Friis, 2010) are known as spikelets (in one species arranged in a compound inflorescence) with spirally arranged pistillate flowers bearing adhering pollen. There is a floral cup that could be interpreted as a perianth tube. Its most conspicuous lobe is abaxial. The ovary is uniovulate and semi-inferior with an ovule that is apparently orthotropous. The overall morphology resembles that of pistillate flowers of *Hedyosmum* (see Kvaček and Friis, 2010; Doyle and Endress, 2018), though the median tepal is adaxial rather than abaxial in *Hedyosmum.* The absence of data on ovule attachment and floral vasculature complicates morphological comparisons of *Zlatkocarpus.*

The fossil *Donlesia* is known as fruits with characteristic long appendages; these fruits are associated with dichotomous leaves that are reportedly whorled (Dilcher and Wang, 2009; Wang and Dilcher, 2018). *Donlesia dakotensis* Dilcher & Wang from the Dakota Formation, late Albian, Kansas, United States (Dilcher and Wang, 2009) differs from extant *Ceratophyllum* in several respects, including basal placentation of the ovule and the occurrence of a long fruit stalk interpreted as a pedicel that could represent a gynophore. The placentation type of *D. dakotensis* has been inferred using embryo orientation alone, but could now be reinterpreted considering the occurrence of atypical ovule positions documented for *Ceratophyllum* in the present study. Another species, *Donlesia cheyennensis*, from even older deposits (Cheyenne Sandstone, early Albian, Kansas), has similar appendages and a stalk, but details of its embryo orientation are unknown (Wang and Dilcher, 2018). There are younger appendaged fossil fruits classified directly in *Ceratophyllum*, such as *C. lesii* Estrada-Ruiz et al. (2009) from the late Campanian of Mexico, which also lacks data on seed orientation. Some fossil long-appendaged diaspores remain problematic with respect to their interpretation and phylogenetic relationships. *Ceratostratiotes sinjanus* (Kerner) Bužek from Early Miocene of Austria has been considered a fruit of Ceratophyllaceae (reviewed by Les, 1988), but could equally be interpreted as an appendaged seed (not a fruit) with morphological similarities to Hydrocharitaceae (Meller and van Bergen, 2003). Dilcher and Wang (2009) have maintained the fruit interpretation for *Ceratostratiotes*, and have provided comparisons with *Donlesia* and *Ceratophyllum*.

### To What Extent Is the Evolution of Syncarpy Homoplastic in Angiosperms?

Ancestral state reconstruction of carpel fusion with parsimony does not provide robust support for apocarpy as the unequivocally ancestral condition for mesangiosperms, a clade that comprises the vast majority of extant angiosperms (Sokoloff et al., 2013a; Massoni, 2014; present study: Figure 13). Moreover, our interpretation of the pistillate flower of *Ceratophyllum* adds weight to the scenario that the common ancestor of mesangiosperms had congenitally united carpels (Figure 13). We explored the potential value of considering only congenital carpel fusion. In theory, rescoring taxa with postgenital fusion as free-carpellate and restricting syncarpy to taxa with congenital fusion could favour scenarios with delayed origins of syncarpy, because more terminals in the data set will be scored as lacking fusion. However, our analyses have revealed that such rescoring has only limited effect. Even when syncarpy is restricted to congenital fusion only, none of the 96 topologies considered in Figure 13 (see also Supplementary File 2) unequivocally suggested ancestral apocarpy for mesangiosperms. When the gynoecium of *Ceratophyllum* is interpreted as pseudomonomerous, most tree topologies unequivocally suggest the occurrence of ancestral syncarpy in mesangiosperms and this result is not sensitive to different interpretations of syncarpy (compare top right and bottom right quarters of Figure 13).

Interestingly, the issue of early evolution of syncarpy in angiosperms is not clarified by the use of sophisticated model-based methods that take into account phylogenetic uncertainty and branch lengths rather than parsimony. Sauquet et al. (2017) found low support for apocarpy as the ancestral state for all angiosperms (with posterior probability 0.73 and its associated credibility interval 0.09–1 inferred from the reversible-jump Bayesian analysis of the series of trees preferred in that study), meaning that the ancestral condition of the gynoecium fusion character would be more safely interpreted as uncertain.

The idea that apocarpy is ancestral for angiosperms is almost universally accepted in the literature of the last half-century. An exception is the work by Haines and Lye (1986), who suggested that apocarpy is of secondary origin wherever it occurs in angiosperms, and that syncarpous gynoecia ripening to loculicidal capsules may represent an ancestral condition. The traditional view that apocarpy is ancestral in angiosperms is apparently based on the assumption that a carpel is a likely megasporophyll homologue and that sporophylls of the hypothetical angiosperm ancestors were free from each other. However, the ancestors remain hypothetical and the homologies of the angiosperm carpel remain problematic (Bateman et al., 2006; Doyle, 2008; Doyle, 2012). Organ fusions are known in reproductive structures of some Bennettitales, Cupressaceae (*Juniperus*) and Gnetales (Mundry and Stützel, 2004; Pott et al., 2017; Popa, 2019). These three gymnosperm groups share whorled organ arrangement in reproductive structures, a condition that facilitates organ fusions in angiosperm flowers (Endress, 1987a; Endress, 2006). It is notable in this context that there is evidence (though statistical support is low) for the ancestrally whorled morphology of angiosperm flowers (Sauquet et al., 2017; Sokoloff et al., 2018a).

Endress (1982) and Armbruster et al. (2002) highlighted the functional advantages of syncarpy as compared with apocarpy. One possible explanation for the observed controversies in reconstructions of gynoecium evolution is that apocarpy is ancestral but many early angiosperm lineages rapidly experienced parallel gains of syncarpy. In the framework of this explanation, transitions were so frequent because of the high adaptive value of syncarpy, including the presence of an internal compitum. In other words, the evolution of syncarpy was highly homoplastic, and scenarios suggested by formal analyses of character evolution could be misleading, especially if only extant representatives are considered. As highlighted by Phillips et al. (2020) the growing amount of data on the potential roles of the *NAM/CUC3* subfamily of *NAC* transcription factors as regulators of floral fusions is highly congruent with the idea of very homoplastic evolution of syncarpy.

A comparison can be made with leaf evolution in tracheophytes. The origin of leaves had such a strong adaptive value that no telomic tracheophytes have survived up to the present time (Harrison and Morris, 2017). Therefore, in the absence of access to the fossil record, it would be difficult to obtain robust evidence for the absence of leaves in the most recent common ancestor of all extant euphyllophytes, and especially to estimate the degree of homoplasy in leaf origins. Testing a similar scenario of multiple repeated gains of syncarpy clearly requires the use of the angiosperm fossil record. Data on well-investigated fossil angiosperm flowers should be incorporated into large-scale analyses of the evolution of floral characters. Current studies widely use fossils to calibrate phylogenetic trees and produce chronograms. An angiosperm-wide data set was used to infer the phylogenetic placements of fossil flowers (Schönenberger et al., 2020). A logical next step is to combine data on fossil floral characters with floral characters of extant taxa to produce refined concepts of morphological evolution. Admittedly, more than one phylogenetic placement is possible for many fossils (e.g. von Balthazar et al., 2008; Doyle and Endress, 2014; Friis et al., 2021). Computational testing of all the combinations of possible placements of well-investigated fossils could help in inferring the ancestral gynoecium type in both mesangiosperms and angiosperms as a whole. The present study provides a methodical example of such an approach for ‘orphan’ taxa (Figure 13).

Developmental genetics represents a complementary means of elucidating patterns of gynoecium evolution in angiosperms. Ontogenetic formation of syncarpous gynoecia exhibits rather complex and diverse combinations of fusion phenomena. Both congenital and postgenital fusions take place during the development of most syncarpous gynoecia, with postgenital fusions playing important roles in closure of the inner space of the gynoecium and formation of the pollen-tube transmitting tissue. The loss of organ boundaries responsible for fusion between carpels could potentially be related to NAM/CUC3 transcription factors (Phillips et al., 2020). However, at the same time, specific boundaries must be patterned in the plurilocular synascidiate zone of the gynoecium and/or the free stigmas. Potential defects in the latter process could result in formation of some reduced types of gynoecia like that most commonly occurring in *Ceratophyllum*.

### Value of Teratology in Evolutionary Morphology

The present study highlights an old problem concerning the value of teratological data in evolutionary morphology. This problem has no general solution and questions of morphological homologies require a case-by-case approach. More lines of evidence allow more robust conclusions to be made, but unfortunately different types of evidence can sometimes be contradictory (e.g., Rutishauser and Sattler, 1987). It is clear that some terata (though not all) can provide useful information on homologies and evolution, but the process of distinguishing homology-informative terata cannot be fully formalized, as modern developmental-geneticists are well aware. A complete ignorance of teratology in evolutionary morphology is problematic already because there is only a quantitative boundary between the norm and abnormalities. What is normal or common in one species may be abnormal in closely related species (Meyen, 1973). Furthermore, the continuum between the norm and anomaly makes analyses of character evolution based on matrices of discrete character states problematic. Model-based methods provide us with estimates of probabilities of the occurrence of certain character states in ancestral nodes, but in reality we may need to think in terms of frequencies of character states in terminal groups and internal nodes of phylogenetic trees.

The present study provides a good example of the elusive nature of distinguishing between the norm and anomaly. In our material, the glandular appendage(s) of the gynoecium is consistently present and represents a norm in *C. demersum*, but is observed only sporadically in its typical form in *C. tanaiticum*. Vestigial gynoecium glands are found in both *C. tanaiticum* and *C. submersum*. Variation in the number of distal gynoecium outgrowths is documented in all three examined species and therefore should be incorporated in the picture of our knowledge on *Ceratophyllum*.

Resolving the problem of the identity of involucral appendages provides an example of the importance of sporadic anomalies. In the past, the involucral appendages were often interpreted as tepals or sepals (Cronquist, 1981; Sehgal and Ram, 1981; Takhtajan, 1987). The most important argument against this view is the sporadic occurrence of branching above the whorl of involucral appendages in the pistillate reproductive units in *C. demersum* (Aboy, 1936). As documented in detail by Aboy (1936), the branches develop next-order reproductive units, each with its own involucre surrounding a gynoecium. This is why the reproductive units of *Ceratophyllum* are interpreted as reduced inflorescences rather than perianth-bearing flowers (Les, 1986; Les, 1993; Endress, 1994; Endress, 2001; Endress and Doyle, 2009; Endress and Doyle, 2015). We support this conclusion, but the findings of Aboy (1936) clearly belong to the area of teratology. Several detailed SEM-based studies of the structure and development of *C. demersum* have been performed after 1936 (Rutishauser and Sattler, 1987; Endress 1994; Endress, 2001; Iwamoto et al., 2003; Iwamoto et al., 2015; the present study), but only one light microscopy work has revealed data on pistillate flowers resembling those of Aboy: Sehgal and Ram (1981) mentioned rare flowers with two free carpels. This observation of Sehgal and Ram (1981) is unfortunately not documented by any photograph or drawing. We do not know whether both pistils were at the same developmental stage and whether any of them had an additional involucre. Mutants of *Arabidopsis* with branched inflorescence-like flowers are well-characterized (Bowman et al., 1993; Kempin et al., 1995) but generally not regarded as evidence for inferring more complex homologies of conventional wild-type flowers of Brassicaceae.

Apart from the terata described by Aboy (1936), the inflorescence interpretation of reproductive units is supported by continuous stamen development in staminate reproductive units of *Ceratophyllum* (Endress, 2004; Endress and Doyle, 2009; Endress and Doyle, 2015). This developmental pattern better fits the idea of a spike with unistaminate naked bractless flowers. Angiosperm flowers that show the degree of unequal maturation that is observed in staminate reproductive units *Ceratophyllum* are apparently unknown (Endress and Doyle, 2015), though this feature could represent a highly specialized pollination mode that is extremely uncommon among angiosperms. Staminate reproductive units of *Hedyosmum* that are morphologically similar to those of *Ceratophyllum* are not characterized by prolonged proliferation (Sokoloff et al., 2018b).

Our data on the pistillate flower of *Ceratophyllum* provide additional arguments in favour of the interpretation of the involucral appendages as extrafloral organs. Indeed, if the ovary is indeed inferior, the involucre cannot be interpreted as a perianth. The present study and that of Aboy (1936) analyse different kinds of developmental abnormality. In isolation, such teratological data are questionable, but taken together they allow an interpretation that is internally consistent and hence plausible.

## Conclusion

The entire structure of the pistillate flower of *Ceratophyllum* shows strong reduction, presumably as a result of a long and complex evolutionary history. Our data indicate that the glandular appendage in the *Ceratophyllum* gynoecium is a reduced tepal or staminode homologue. Therefore, the ovary is inferior, as in most Chloranthaceae and in the potentially related fossils *Canrightia* and *Canrightiopsis*. Our data support the view that the ancestors of extant *Ceratophyllum* had non-monomerous, syncarpous gynoecia. Comparative morphology suggests that the gynoecia of Chloranthaceae and *Ceratophyllum* only mimic solitary ascidiate carpels, in which case their resemblance to individual carpels in taxa such as *Amborella* would be an example of evolutionary convergence. This conclusion has implications for understanding the origin and early evolution of the angiosperm gynoecium.

Our study provides novel arguments in favour of the morphological similarity between *Ceratophyllum* and Chloranthaceae (Endress and Doyle, 2009; Endress and Doyle, 2015; Doyle and Endress, 2018). Recent nuclear phylogenomic studies have tended to reject a sister-group relationship between *Ceratophyllum* and Chloranthaceae (One Thousand Plant Transcriptomes Initiative, 2019; Guo et al., 2021). However, even if these phylogenomic data are confirmed, this will in no way reduce the significance of morphological similarity between the two groups. Instead, it is possible that *Ceratophyllum* and Chloranthaceae are two isolated groups that have retained some important morphological features of a common ancestor of mesangiosperms. Indeed, it is difficult to explain their shared characters through parallel evolution because of the strong ecological differences between *Ceratophyllum* and Chloranthaceae.

The widely accepted notion that apocarpy is ancestral in both mesangiosperms and angiosperms in general lacks robust support, even using recent phylogenetic data. Resolving the problem of inferring the early evolution of the angiosperm gynoecium lies in interpretation of the available morphological data and accumulation of deeper and wider morphological knowledge, rather than in refining computational methods of ancestral character reconstruction. Further progress in large-scale studies of evolution of floral characters, including the gynoecium, would benefit not only from developmental genetic, broad-scale genomic and more targeted gene approaches, but also from improved morphological data sets that include characters of fossil flowers.

## Data Availability

The original contributions presented in the study are included in the article/Supplementary Material, further inquiries can be directed to the corresponding author.
