# Supplementary material for: Refined Interpretation of the Pistillate Flower in Ceratophyllum Sheds Fresh Light on Gynoecium Evolution in Angiosperms
Source: Front Cell Dev Biol. 2022 Apr 28;10:868352. doi: 10.3389/fcell.2022.868352 (PMC9098228; doi:10.3389/fcell.2022.868352)
Supplement: Supplementary file 1 [file DataSheet2.PDF]

Online supplementary file 2. Ancestral state reconstructions with parsimony for carpel fusion in mesangiosperms.

The tables show to what extent reconstructions of gynoecium evolution in angiosperms are sensitive to contrasting interpretations of the female flower in *Ceratophyllum*.

Table S1. Reconstructions of the ancestral type of gynoecium in mesangiosperms under different topologies of phylogenetic trees (Fig. 2B). Relative positions of monocots, eudicots, Chloranthaceae and magnoliids are fixed here as in the **JM tree** (Fig. 2A). Original data set from Sauquet *et al.* (2017) is used, character 403_A: Fusion of ovaries (binary). This character makes **no difference between congenital and postgenital fusion**. Each cell in the table is the reconstructed ancestral condition for mesangiosperms (present = fusion between carpels is present). Before a slash in each cell is what is found when *Ceratophyllum* is re-scored as having fused carpels when its gynoecium is interpreted as pseudomonomerous. After a slash is what is found when *Ceratophyllum* is scored as unknown (as in Sauquet *et al.*, 2017), a scoring that is necessary if it has only one carpel (monomery).

		Euptelea sister to all other Ranunculales		*Euptelea* sister to Papaveraceae	
		Araceae sister to all other Alismatales	Tofieldiaceae sister to all other Alismatales	Araceae sister to all other Alismatales	Tofieldiaceae sister to all other Alismatales
Ceratophyllum sister to Chloranthaceae	*Nuphar* + Nymphaeoideae	Present/ Uncertain	Present/ Uncertain	Present/ Uncertain	Present/ Uncertain
	Nuphar + Cabombaceae	Present/ Uncertain	Present/ Uncertain	Present/ Uncertain	Present/ Uncertain
Ceratophyllum sister to eudicots	*Nuphar* + Nymphaeoideae	Present/ Uncertain	Present/ Uncertain	Present/ Uncertain	Present/ Uncertain
	Nuphar + Cabombaceae	Present/ Uncertain	Present/ Uncertain	Present/ Uncertain	Present/ Uncertain
Ceratophyllum sister to monocots	*Nuphar* + Nymphaeoideae	Present/ Uncertain	Present/ Uncertain	Present/ Uncertain	Present/ Uncertain
	Nuphar + Cabombaceae	Present/ Uncertain	Present/ Uncertain	Present/ Uncertain	Present/ Uncertain
Ceratophyllum sister to other mesangiosperms	*Nuphar* + Nymphaeoideae	Present/ Uncertain	Present/ Uncertain	Present/ Uncertain	Present/ Uncertain
	Nuphar + Cabombaceae	Present/ Uncertain	Present/ Uncertain	Present/ Uncertain	Present/ Uncertain

Table S2. Reconstructions of the ancestral type of gynoecium in mesangiosperms under different topologies of phylogenetic trees (Fig. 2B). Relative positions of monocots, eudicots, Chloranthaceae and magnoliids are fixed here as in the **JM tree** (Fig. 2A). Our edited version of the gynoecium fusion character is used. It takes into account only **congenital fusion** between carpels. Each cell in the table is the reconstructed ancestral condition for mesangiosperms (present = fusion between carpels is present). Before a slash in each cell is what is found when *Ceratophyllum* is re-scored as having fused carpels when its gynoecium is interpreted as pseudomonomerous. After a slash is what is found when *Ceratophyllum* is scored as unknown (as in Sauquet *et al.*, 2017), a scoring that is necessary if it has only one carpel (monomery).

		Euptelea sister to all other Ranunculales		*Euptelea* sister to Papaveraceae	
		Araceae sister to all other Alismatales	Tofieldiaceae sister to all other Alismatales	Araceae sister to all other Alismatales	Tofieldiaceae sister to all other Alismatales
Ceratophyllum sister to Chloranthaceae	*Nuphar* + Nymphaeoideae	Present/ Uncertain	Present/ Uncertain	Present/ Uncertain	Present/ Uncertain
	Nuphar + Cabombaceae	Present/ Uncertain	Present/ Uncertain	Present/ Uncertain	Present/ Uncertain
Ceratophyllum sister to eudicots	*Nuphar* + Nymphaeoideae	Present/ Uncertain	Uncertain/ Uncertain	Uncertain/ Uncertain	Uncertain/ Uncertain
	Nuphar + Cabombaceae	Present/ Uncertain	Uncertain/ Uncertain	Uncertain/ Uncertain	Uncertain/ Uncertain
Ceratophyllum sister to monocots	*Nuphar* + Nymphaeoideae	Uncertain/ Uncertain	Uncertain/ Uncertain	Uncertain/ Uncertain	Uncertain/ Uncertain
	Nuphar + Cabombaceae	Uncertain/ Uncertain	Uncertain/ Uncertain	Uncertain/ Uncertain	Uncertain/ Uncertain
Ceratophyllum sister to other mesangiosperms	*Nuphar* + Nymphaeoideae	Present/ Uncertain	Present/ Uncertain	Present/ Uncertain	Present/ Uncertain
	Nuphar + Cabombaceae	Present/ Uncertain	Present/ Uncertain	Present/ Uncertain	Present/ Uncertain

Table S3. Reconstructions of the ancestral type of gynoecium in mesangiosperms under different topologies of phylogenetic trees (Fig. 2B). Relative positions of monocots, eudicots, Chloranthaceae and magnoliids are fixed here as in the **1KP tree** (Fig. 2A). Original data set from Sauquet *et al.* (2017) is used, character 403_A: Fusion of ovaries (binary). This character makes **no difference between congenital and postgenital fusion**. Each cell in the table is the reconstructed ancestral condition for mesangiosperms (present = fusion between carpels is present). Before a slash in each cell is what is found when *Ceratophyllum* is re-scored as having fused carpels when its gynoecium is interpreted as pseudomonomerous. After a slash is what is found when *Ceratophyllum* is scored as unknown (as in Sauquet *et al.*, 2017), a scoring that is necessary if it has only one carpel (monomery).

		Euptelea sister to all other Ranunculales		*Euptelea* sister to Papaveraceae	
		Araceae sister to all other Alismatales	Tofieldiaceae sister to all other Alismatales	Araceae sister to all other Alismatales	Tofieldiaceae sister to all other Alismatales
Ceratophyllum sister to Chloranthaceae	*Nuphar* + Nymphaeoideae	Present/ Uncertain	Present/ Uncertain	Present/ Present	Present/ Present
	Nuphar + Cabombaceae	Present/ Uncertain	Present/ Uncertain	Present/ Present	Present/ Present
Ceratophyllum sister to eudicots	*Nuphar* + Nymphaeoideae	Present/ Uncertain	Present/ Uncertain	Present/ Present	Present/ Present
	Nuphar + Cabombaceae	Present/ Uncertain	Present/ Uncertain	Present/ Present	Present/ Present
Ceratophyllum sister to monocots	*Nuphar* + Nymphaeoideae	Uncertain/ Uncertain	Uncertain/ Uncertain	Present/ Present	Present/ Present
	Nuphar + Cabombaceae	Uncertain/ Uncertain	Uncertain/ Uncertain	Present/ Present	Present/ Present
Ceratophyllum sister to other mesangiosperms	*Nuphar* + Nymphaeoideae	Present/ Uncertain	Present/ Uncertain	Present/ Uncertain	Present/ Uncertain
	Nuphar + Cabombaceae	Present/ Uncertain	Present/ Uncertain	Present/ Uncertain	Present/ Uncertain

Table S4. Reconstructions of the ancestral type of gynoecium in mesangiosperms under different topologies of phylogenetic trees (Fig. 2B). Relative positions of monocots, eudicots, Chloranthaceae and magnoliids are fixed here as in the **1KP tree** (Fig. 2A). Our edited version of the gynoecium fusion character is used. It takes into account only **congenital fusion** between carpels. Each cell in the table is the reconstructed ancestral condition for mesangiosperms (present = fusion between carpels is present). Before a slash in each cell is what is found when *Ceratophyllum* is re-scored as having fused carpels when its gynoecium is interpreted as pseudomonomerous. After a slash is what is found when *Ceratophyllum* is scored as unknown (as in Sauquet *et al.*, 2017), a scoring that is necessary if it has only one carpel (monomery).

		Euptelea sister to all other Ranunculales		*Euptelea* sister to Papaveraceae	
		Araceae sister to all other Alismatales	Tofieldiaceae sister to all other Alismatales	Araceae sister to all other Alismatales	Tofieldiaceae sister to all other Alismatales
Ceratophyllum sister to Chloranthaceae	*Nuphar* + Nymphaeoideae	Present/ Uncertain	Present/ Uncertain	Present/ Present	Present/ Present
	Nuphar + Cabombaceae	Present/ Uncertain	Present/ Uncertain	Present/ Present	Present/ Present
Ceratophyllum sister to eudicots	*Nuphar* + Nymphaeoideae	Present/ Uncertain	Present/ Uncertain	Present/ Present	Present/ Present
	Nuphar + Cabombaceae	Present/ Uncertain	Present/ Uncertain	Present/ Present	Present/ Present
Ceratophyllum sister to monocots	*Nuphar* + Nymphaeoideae	Uncertain/ Uncertain	Uncertain/ Uncertain	Present/ Present	Present/ Present
	Nuphar + Cabombaceae	Uncertain/ Uncertain	Uncertain/ Uncertain	Present/ Present	Present/ Present
Ceratophyllum sister to other mesangiosperms	*Nuphar* + Nymphaeoideae	Present/ Uncertain	Present/ Uncertain	Present/ Uncertain	Present/ Uncertain
	Nuphar + Cabombaceae	Present/ Uncertain	Present/ Uncertain	Present/ Uncertain	Present/ Uncertain

Table S5. Reconstructions of the ancestral type of gynoecium in mesangiosperms under different topologies of phylogenetic trees (Fig. 2B). Relative positions of monocots, eudicots, Chloranthaceae and magnoliids are fixed here as in the **DE tree** (Fig. 2A). Original data set from Sauquet *et al.* (2017) is used, character 403_A: Fusion of ovaries (binary). This character makes **no difference between congenital and postgenital fusion**. Each cell in the table is the reconstructed ancestral condition for mesangiosperms (present = fusion between carpels is present). Before a slash in each cell is what is found when *Ceratophyllum* is re-scored as having fused carpels when its gynoecium is interpreted as pseudomonomerous. After a slash is what is found when *Ceratophyllum* is scored as unknown (as in Sauquet *et al.*, 2017), a scoring that is necessary if it has only one carpel (monomery).

		Euptelea sister to all other Ranunculales		*Euptelea* sister to Papaveraceae	
		Araceae sister to all other Alismatales	Tofieldiaceae sister to all other Alismatales	Araceae sister to all other Alismatales	Tofieldiaceae sister to all other Alismatales
Ceratophyllum sister to Chloranthaceae	*Nuphar* + Nymphaeoideae	Present/ Uncertain	Present/ Uncertain	Present/ Uncertain	Present/ Uncertain
	Nuphar + Cabombaceae	Present/ Uncertain	Present/ Uncertain	Present/ Uncertain	Present/ Uncertain
Ceratophyllum sister to eudicots	*Nuphar* + Nymphaeoideae	Uncertain/ Uncertain	Uncertain/ Uncertain	Uncertain/ Uncertain	Uncertain/ Uncertain
	Nuphar + Cabombaceae	Uncertain/ Uncertain	Uncertain/ Uncertain	Uncertain/ Uncertain	Uncertain/ Uncertain
Ceratophyllum sister to monocots	*Nuphar* + Nymphaeoideae	Uncertain/ Uncertain	Uncertain/ Uncertain	Uncertain/ Uncertain	Uncertain/ Uncertain
	Nuphar + Cabombaceae	Uncertain/ Uncertain	Uncertain/ Uncertain	Uncertain/ Uncertain	Uncertain/ Uncertain
Ceratophyllum sister to other mesangiosperms	*Nuphar* + Nymphaeoideae	Present/ Uncertain	Present/ Uncertain	Present/ Uncertain	Present/ Uncertain
	Nuphar + Cabombaceae	Present/ Uncertain	Present/ Uncertain	Present/ Uncertain	Present/ Uncertain

Table S6. Reconstructions of the ancestral type of gynoecium in mesangiosperms under different topologies of phylogenetic trees (Fig. 2B). Relative positions of monocots, eudicots, Chloranthaceae and magnoliids are fixed here as in the **DE tree** (Fig. 2A). Our edited version of the gynoecium fusion character is used. It takes into account only **congenital fusion** between carpels. Each cell in the table is the reconstructed ancestral condition for mesangiosperms (present = fusion between carpels is present). Before a slash in each cell is what is found when *Ceratophyllum* is re-scored as having fused carpels when its gynoecium is interpreted as pseudomonomerous. After a slash is what is found when *Ceratophyllum* is scored as unknown (as in Sauquet *et al.*, 2017), a scoring that is necessary if it has only one carpel (monomery).

		Euptelea sister to all other Ranunculales		*Euptelea* sister to Papaveraceae	
		Araceae sister to all other Alismatales	Tofieldiaceae sister to all other Alismatales	Araceae sister to all other Alismatales	Tofieldiaceae sister to all other Alismatales
Ceratophyllum sister to Chloranthaceae	*Nuphar* + Nymphaeoideae	Present/ Uncertain	Present/ Uncertain	Present/ Uncertain	Present/ Uncertain
	Nuphar + Cabombaceae	Present/ Uncertain	Present/ Uncertain	Present/ Uncertain	Present/ Uncertain
Ceratophyllum sister to eudicots	*Nuphar* + Nymphaeoideae	Uncertain/ Uncertain	Uncertain/ Uncertain	Uncertain/ Uncertain	Uncertain/ Uncertain
	Nuphar + Cabombaceae	Uncertain/ Uncertain	Uncertain/ Uncertain	Uncertain/ Uncertain	Uncertain/ Uncertain
Ceratophyllum sister to monocots	*Nuphar* + Nymphaeoideae	Uncertain/ Uncertain	Uncertain/ Uncertain	Uncertain/ Uncertain	Uncertain/ Uncertain
	Nuphar + Cabombaceae	Uncertain/ Uncertain	Uncertain/ Uncertain	Uncertain/ Uncertain	Uncertain/ Uncertain
Ceratophyllum sister to other mesangiosperms	*Nuphar* + Nymphaeoideae	Present/ Uncertain	Present/ Uncertain	Present/ Uncertain	Present/ Uncertain
	Nuphar + Cabombaceae	Present/ Uncertain	Present/ Uncertain	Present/ Uncertain	Present/ Uncertain
