# Supplementary material for: Refined Interpretation of the Pistillate Flower in Ceratophyllum Sheds Fresh Light on Gynoecium Evolution in Angiosperms
Source: Front Cell Dev Biol. 2022 Apr 28;10:868352. doi: 10.3389/fcell.2022.868352 (PMC9098228; doi:10.3389/fcell.2022.868352)
Supplement: Supplementary file 2 [file DataSheet3.PDF]

Online Supplementary File 3. Major floral characters of selected fossil and extant taxa potentially related to *Ceratophyllum* (Endress and Doyle, 2015; Friis et al., 2015; Kvaček et al., 2016; Gomez et al., 2020; this study).

Taxon and age	*Ceratophyllum* extant	*Pseudoasterophyllites* Cenomanian	*Montsechia* Barremian	*Canrightia* Barremian to Albian	*Zlatkocarpus* Cenomanian	*Canrightiopsis* Albian	*Hedyosmum* extant	*Ascarina* extant	*Chloranthus* and *Sarcandra* extant
Flower gender	male and female	male and female	stamens unknown	bisexual	stamens unknown	bisexual	male and female	male and female	bisexual
Tepals	possible rudiment in female flower	absent	not found	present in a reduced form	present	absent	present in female flowers	absent	absent
Stamens per flower	1	1	unknown	about 4	unknown	3	1	1-few	1***
Pistils per flower	1	1	1 (or 2**)	1	1	1	1	1	1
Ovary position	inferior*	uncertain	uncertain	semi-inferior	semi-inferior	semi-inferior	inferior	uncertain	semi-inferior
Ovary locules	1	1	1	1	1	1	1	1	1
Ovules per ovary	1	1	1	2-5	1	1	1	1	1
Ovule position	usually pendent	pendent	pendent	pendent	unknown	pendent	pendent	pendent	pendent
Ovule orthotropous	yes	yes	yes	yes	?yes	yes	yes	yes	yes
Integuments per ovule	?1	?	1	2	?	2	2	2	2

* Our preferred interpretation.

** Depending on interpretation.

*** Possibly a fusion product of three stamens in *Chloranthus*.
