# Supplementary material for: Refined Interpretation of the Pistillate Flower in Ceratophyllum Sheds Fresh Light on Gynoecium Evolution in Angiosperms
Source: Front Cell Dev Biol. 2022 Apr 28;10:868352. doi: 10.3389/fcell.2022.868352 (PMC9098228; doi:10.3389/fcell.2022.868352)
Supplement: Supplementary file 3 [file DataSheet1.PDF]

Online Supplement 1. Morphological data set used in the paper.

In all characters, 0 = fusion between carpels is present; 1 = fusion between carpels is absent
Character A: the oririnal character 403 A from Sauquet et al. (2017). All types of fusion are considered. The character is scored as uncertain in Ceratophyllum.
Character B: the same as Character A, but Ceratophyllum is scored as having fused carpels.
Character C: modified character 403 A from Sauquet et al. (2017). Only congenital fusion is considered. The character is scored as uncertain in Ceratophyllum.
Character D: the same as Character C, but Ceratophyllum is scored as having fused carpels.

**Taxon**	**Character**				**Comments**
	A	B	C	D	
Abatia parviflora	1	1	1	1	
Abelia triflora	?	?	1	1	Abelia has an inferior ovary with a fertile and two sterile ovary locules (Qiner & Landrein in Flora of China, vol. 19), thus the gynoecium is syncarpous, postgenital fusions are not reported from Dipsacales
Abrophyllum ornans	1	1	1	1	
Acacia fimbriata	?	?	?	?	
Acalypha californica	?	?	?	?	
Acanthochlamys bracteata	1	1	1	1	
Acanthus montanus	1	1	1	1	
Acharia tragodes	1	1	1	1	
Acicarpha tribuloides	1	1	1	1	
Acorus gramineus	1	1	1	1	
Acridocarpus natalitius	?	?	?	?	
Actinidia chinensis	1	1	1	1	
Adoxa moschatellina	1	1	1	1	
Aesculus pavia	1	1	1	1	
Aextoxicon punctatum	?	?	?	?	
Afrolicania elaeosperma	?	?	?	?	
Afrostyrax lepidophyllus	1	1	1	1	
Agave ghiesbreghtii	?	?	?	?	
Ailanthus altissima	?	?	0	0	Endress et al. 1983, Nord. J. Bot. 3: 293-300.
Akebia quinata	0	0	0	0	
Alangium chinense	?	?	?	?	

Albizia julibrissin	?	?	?	?	
Alisma plantago-aquatica	0	0	0	0	
Allium fistulosum	1	1	1	1	
Alluaudia procera	1	1	1	1	
Alnus alnobetula	1	1	1	1	
Alophia drummondii	1	1	1	1	
Alseuosmia macrophylla	?	?	?	?	
Alstroemeria aurea	?	?	1	1	Buxbaum 1954, Österr. Bot. Zeitschr. 101: 337–352 (A. aurantiaca)
Altingia excelsa	?	?	1	1	Bogle 1986, Ann. Missouri Bot. Gard. 73: 325-347.
Amborella trichopoda	0	0	0	0	
Anagallis tenella	1	1	1	1	
Anarthria polyphylla	1	1	1	1	
Ancistrocladus korupensis	1	1	1	1	
Androsace spinulifera	1	1	1	1	
Androstachys johnsonii	1	1	1	1	
Anemopsis californica	?	?	1	1	Endress and Igersheim 1998, Bot. J. Linn. Soc. 127: 289-370.
Anethum graveolens	1	1	1	1	
Aneulophus africanus	1	1	1	1	
Angelica sylvestris	?	?	1	1	Gynoecium is syncarpous and bicarpellate in Apiaceae. Angelica sylvestris: e.g., Tikhomirov 1958, Nauchn. Dokl. Vysshey Shkoly, Biol. Nauki (1): 129-138
Anigozanthos flavidus	?	?	1	1	Simpson 1993, Syst. Bot. 18: 593–613.
Anisophyllea fallax	?	?	1	1	Matthews et al.2001, Ann. Bot. 88: 439-455 (Anisophyllea disticha)
Anisoptera marginata	1	1	1	1	
Annona muricata	?	?	0	0	carpels are postgenitally united, Igersheim and Endress, 1997, Bot. J. Linn. Soc. 124: 213-271.
Anopterus macleayanus	1	1	1	1	
Anredera cordifolia	1	1	1	1	
Antirrhinum majus	1	1	1	1	
Aphanopetalum resinosum	1	1	1	1	

Aphloia theiformis	?	?	?	?	
Apium graveolens	1	1	1	1	
Aporosa frutescens	1	1	1	1	
Arabidopsis thaliana	1	1	1	1	
Aralia spinosa	?	?	1	1	Erbar and Leins 1998, Flora 180: 391-406 (Aralia elata)
Aralidium pinnatifidum	1	1	1	1	
Arbutus unedo	1	1	1	1	
Archytaea triflora	1	1	1	1	
Arctopus echinatus	1	1	1	1	
Argophyllum laxum	1	1	1	1	
Aristea glauca	1	1	1	1	
Aristolochia macrophylla	1	1	1	1	
Asarum canadense	?	?	1	1	Igersheim and Endress 1998, Bot. J. Linn. Soc. 127: 289-370 (Asarum).
Ascarina lucida	?	?	?	?	
Asimina triloba	?	?	0	0	Igersheim and Endress 1997, Bot. J. Linn. Soc. 124: 213-271.
Asparagus officinalis	1	1	1	1	
Asteropeia micraster	1	1	1	1	
Astragalus membranaceus	?	?	?	?	
Atherosperma moschatum	0	0	0	0	
Atropa belladonna	1	1	1	1	
Atuna racemosa	?	?	?	?	
Aucuba japonica	?	?	?	?	
Austrobaileya scandens	0	0	0	0	
Austrobuxus megacarpus	1	1	1	1	
Averrhoa carambola	1	1	1	1	
Azorella selago	?	?	1	1	Liu et al. 2009, Plant Syst. Evol. 280: 1-13 (Azorella fruits)
Balanops vieillardii	1	1	1	1	
Baloskion tetraphyllum	?	?	1	1	Kircher 1986, Dissert. Bot. 94: 1-219.
Barbeuia madagascariensis	1	1	1	1	

Barbeya oleoides	?	?	?	?	
Barleria prionitis	?	?	?	?	
Barnadesia caryophylla	?	?	?	?	
Barringtonia asiatica	1	1	1	1	
Basella alba	?	?	1	1	Sattler and Lacroix 1988, Amer. J. Bot. 75: 918-927 (our interpretation of data presented in this paper)
Batis maritima	1	1	1	1	
Bauhinia galpinii	?	?	?	?	
Beaucarnea recurvata	1	1	?	?	potential roles of postgenital and congenital fusions in gynoecium development are unclear
Begonia sanguinea	1	1	1	1	
Berberidopsis corallina	1	1	1	1	
Bergia texana	1	1	1	1	
Berzelia lanuginosa	?	?	?	?	
Beta vulgaris	1	1	1	1	
Bhesa paniculata	1	1	1	1	
Biebersteinia orphanidis	1	1	?	?	potential roles of postgenital and congenital fusions in gynoecium development are unclear
Billardiera heterophylla	1	1	1	1	
Bischofia javanica	1	1	1	1	
Bixa orellana	1	1	1	1	
Blandfordia punicea	1	1	1	1	
Boehmeria nivea	?	?	?	?	
Bomarea edulis	1	1	1	1	
Bombax ceiba	1	1	1	1	
Bonnetia sessilis	1	1	1	1	
Boopis graminea	1	1	1	1	
Borago officinalis	1	1	1	1	
Borya septentrionalis	1	1	0	0	Rudall 2002, Int. J. Plant Sci. 163: 261–276.
Bougainvillea glabra	?	?	?	?	
Brasenia schreberi	0	0	0	0	
Brassica napus	1	1	1	1	
Brexia madagascariensis	1	1	1	1	
Bruguiera gymnorhiza	1	1	1	1	
Brunellia acutangula	0	0	0	0	
Brunia albiflora	1	1	1	1	

Bulbine succulenta	1	1	1	1	
Burmannia biflora	1	1	1	1	
Bursera fagaroides	1	1	1	1	
Buxus sempervirens	1	1	1	1	
Byblis liniflora	1	1	1	1	
Byrsonima crassifolia	1	1	1	1	
Cabomba caroliniana	0	0	0	0	
Calceolaria integrifolia	1	1	1	1	
Calectasia intermedia	1	1	1	1	
Callicarpa dichotoma	?	?	?	?	
Calophyllum soulattri	?	?	?	?	
Calycanthus floridus	0	0	0	0	
Camellia sinensis	1	1	1	1	
Campanula elatines	1	1	1	1	
Campanula trachelium	1	1	1	1	
Campsis radicans	1	1	1	1	
Camptotheca acuminata	?	?	?	?	
Campynema lineare	1	1	1	1	
Cananga odorata	?	?	0	0	Swamy 1956, J. Arnold Arbor. 37: 366-372.
Canella winterana	1	1	1	1	
Canna indica	1	1	1	1	
Cannabis sativa	1	1	1	1	
Capparis spinosa	?	?	1	1	Naghiloo et al. 2015, Nord. J. Bot. 33: 754–760
Carallia brachiata	?	?	?	?	
Cardiopteris quinqueloba	1	1	1	1	
Carica papaya	1	1	1	1	
Carludovica palmata	?	?	1	1	Harling et al. 1998 in Kubitzki, Families and genera..., 3: 202-215.
Carpodetus serratus	1	1	1	1	
Caryocar glabrum	?	?	?	?	
Caryota mitis	?	?	1	1	Rudall et al. 2001, Int. J. Plant Sci. 172: 674-690.

Casearia sylvestris	1	1	1	1	
Cassine orientalis	?	?	?	?	
Cassipourea lanceolata	1	1	1	1	
Casuarina cunninghamiana	?	?	1	1	Flores and Moseley 1982, Amer. J. Bot. 69: 1673-1684 (C. verticillata)
Catalpa speciosa	?	?	1	1	Chen et al. 2009, Acta Horticult. Sin. 36: 285-290.
Caulophyllum thalictroides	?	?	?	?	
Ceanothus sanguineus	?	?	?	?	
Celastrus scandens	1	1	1	1	
Celosia argentea	?	?	1	1	Payer J. B. 1857. Traite d'organogenie comparee de la fleur (C. margaritacea)
Celtis yunnanensis	?	?	1	1	Omori and Terabayashi, 1993, J. Plant Res. 106 : 249-258 (Celtis)
Centranthus ruber	1	1	1	1	
Centrolepis strigosa	?	?	1	1	Sokoloff et al. 2015, Amer. J. Bot. 102: 1219-1249.
Centroplacus glaucinus	1	1	1	1	
Cephalotus follicularis	0	0	0	0	
Ceratonia siliqua	?	?	?	?	
Ceratophyllum demersum	?	1	?	1	
Cercidiphyllum japonicum	?	?	?	?	
Cercis canadensis	?	?	?	?	
Cespedesia spathulata	1	1	1	1	
Chamaedorea seifrizii	?	?	1	1	Askgaard et al. 2008, An. Jard. Bot. Madrid 65: 197-210 (Chamaedorea)
Chimonanthus praecox	0	0	0	0	
Chloranthus japonicus	?	?	?	?	
Choristylis rhamnoides	1	1	1	1	
Chrysobalanus icaco	1	1	1	1	
Chrysolepis sempervirens	?	?	?	?	
Cicer arietinum	?	?	?	?	
Cichorium intybus	1	1	1	1	

Cinnamodendron ekmanii	1	1	1	1	
Cinnamomum camphora	?	?	?	?	
Circaeaster agrestis	?	?	0	0	when there are 2 carpels, they are free, e.g., Takhtajan 1980, Zhizn' Rasteniy, vol. 5, part 1.
Cissampelos pareira	?	?	?	?	
Citronella suaveolens	1	1	1	1	
Citrus paradisi	?	?	1	1	Payer J. B. 1857. Traite d'organogenie comparee de la fleur (Citrus)
Clarkia xantiana	1	1	1	1	
Clavija eggersiana	1	1	1	1	
Claytonia virginica	1	1	1	1	
Clethra alnifolia	1	1	1	1	
Clidemia petiolaris	1	1	1	1	
Clusia gundlachii	1	1	1	1	
Clutia pulchella	1	1	1	1	
Cobaea scandens	1	1	1	1	
Coccinia sessilifolia	?	?	?	?	
Cocculus orbiculatus	0	0	0	0	
Codiaeum peltatum	?	?	?	?	
Coffea arabica	?	?	1	1	inferior 2-locular ovary in Coffea; no evidence of postgenital fusion of carpels in Rubiaceae: e.g., Igersheim et al. 1994, Bot. Jarhb. Syst. 116: 401-414.
Colchicum speciosum	?	?	1	1	Nordenstam 1998, in Kubitzki, Families and genera..., 3: 175-185
Columellia oblonga	1	1	1	1	
Conceveiba martiana	1	1	1	1	
Connarus championii	0	0	0	0	
Corbichonia decumbens	?	?	?	?	
Coriandrum sativum	?	?	1	1	Gynoecium is syncarpous and bicarpellate in Apiaceae. Coriandrum sativum: Jackson 1933, Amer. J. Bot. 20: 121-144.
Coriaria ruscifolia	?	?	?	?	
Cornus mas	1	1	1	1	
Corokia cotoneaster	?	?	?	?	
Corylopsis pauciflora	?	?	1	1	Endress 1993, in Kubitzki, Families and genera... 3: 322-331 (Corylopsis)

Corynocarpus laevigatus	0	0	?	?	it is unclear whether the gynoecium is monomerous or pseudomonomerous, Matthews and Endress 2004, Bot. J. Linn. Soc. 145: 129–185.
Coula edulis	1	1	1	1	
Couroupita guianensis	1	1	1	1	
Crassula rupestris	0	0	0	0	
Cratoxylum cochinchinense	1	1	1	1	
Crinodendron hookerianum	1	1	1	1	
Crinum asiaticum	1	1	1	1	
Crispiloba disperma	1	1	1	1	
Croizatia brevipetiolata	?	?	?	?	
Croomia pauciflora	?	?	?	?	
Crossosoma bigelovii	0	0	0	0	
Crossostylis grandiflora	1	1	1	1	
Croton alabamensis	?	?	?	?	
Crypteronia paniculata	1	1	1	1	
Cryptocarya meissneriana	?	?	?	?	
Ctenolophon englerianus	?	?	?	?	
Cucumis melo	1	1	1	1	
Cucurbita pepo	1	1	1	1	
Cupaniopsis anacardioides	1	1	?	?	not enough data
Curtisia dentata	1	1	1	1	
Cuscuta cuspidata	1	1	1	1	
Cussonia spicata	?	?	1	1	Eyde, Tseng, 1971, J. Arnold Arbor. 52: 205-239 (C. paniculata).
Cuttsia viburnea	?	?	1	1	Gustafsson 2007, in Kubitzki, Families and genera..., 8: 57-60.
Cyperus alternifolius	?	?	1	1	we follow the interpretation of the gynoecium of Cyperaceae as 2-3-carpellate, e.g. Reynders et al. 2012, Plant Ecol. Evol. 145: 96-125
Cyphia elata	1	1	1	1	
Cypripedium calceolus	1	1	1	1	
Cyrilla racemiflora	1	1	1	1	
Dalechampia spathulata	?	?	?	?	
Dampiera spicigera	?	?	?	?	

Danthonia spicata	?	?	1	1	we follow the interpretation of the gynoecium of Poaceae as pseudomonomerous, e.g. Philipson 1985, Amer. J. Bot. 72: 1954-1961.
Dapania racemosa	1	1	1	1	
Daphnandra micrantha	0	0	0	0	
Daphniphyllum macropodum	1	1	1	1	
Dasypogon bromeliifolius	1	1	1	1	
Datisca cannabina	1	1	1	1	
Daucus carota	?	?	1	1	e.g., Endress 1982, Taxon 31: 48-52.
Davidsonia pruriens	1	1	1	1	
Decaisnea fargesii	0	0	0	0	
Degeneria vitiensis	?	?	0	0	when there are 2 carpels, they are free, e.g., Takhtajan 1980, Zhizn' Rasteniy, vol. 5, part 1.
Delarbrea michieana	?	?	1	1	Eyde, Tseng, 1971, J. Arnold Arbor. 52: 205-239.
Delosperma echinatum	1	1	1	1	
Dendrosicyos socotrana	1	1	1	1	
Denhamia celastroides	1	1	1	1	
Desfontainia spinosa	1	1	1	1	
Dialypetalum floribundum	1	1	1	1	
Dicella nucifera	1	1	1	1	
Dicentra eximia	1	1	1	1	
Dichapetalum rugosum	?	?	?	?	
Didymeles perrieri	?	?	?	?	
Diervilla sessilifolia	1	1	1	1	
Dillenia retusa	?	?	1	1	Endress 2014, Bot. J. Linn. Soc. 174: 1–43 (Dillenia)
Dioscorea alata	?	?	1	1	M.V. Remizowa, pers. obs. for Dioscorea tokoro
Diospyros lotus	1	1	1	1	
Dipelta yunnanensis	1	1	1	1	
Dipentodon sinicus	1	1	1	1	
Dipsacus sativus	?	?	?	?	
Dirachma socotrana	?	?	1	1	Ronse De Craene et al. 2004, Plant Syst. Evol. 249: 111–127

Disanthus cercidifolius	?	?	1	1	Endress 1993, in Kubitzki, Families and genera... 3: 322-331
Dissiliaria muelleri	1	1	1	1	
Donatia fascicularis	?	?	?	?	
Doryphora sassafras	?	?	0	0	Endress 1972, Bot. Jahrb. Syst. 92: 331-428.
Dovyalis rhamnoides	?	?	?	?	
Drimys winteri	0	0	0	0	
Drosera capensis	1	1	1	1	
Drosophyllum lusitanicum	1	1	1	1	
Drypetes madagascariensis	1	1	1	1	
Dudleya viscida	?	?	?	?	
Durandea pentagyna	1	1	1	1	
Durio zibethinus	?	?	1	1	Honsho et al. 2004, J. Amer. Soc. Hort. Sci. 129: 42-45.
Ecdeiocolea monostachya	1	1	1	1	
Echinops exaltatus	1	1	1	1	
Ehretia acuminata	?	?	1	1	Zhu et al., 1995, Flora of China, vol. 16: common style
Elaeagnus umbellata	?	?	?	?	
Elaeis guineensis	1	1	1	1	
Elaeocarpus reticulatus	1	1	1	1	
Elatine triandra	1	1	1	1	
Eliea articulata	1	1	1	1	
Endospermum moluccanum	1	1	1	1	
Enkianthus campanulatus	?	?	1	1	Kosugi 1954, J. Jap. Soc. Horticult. Sci. 23: 53-56 (E. perulatus).
Eremosyne pectinata	1	1	1	1	
Eriocaulon aquaticum	1	1	1	1	
Erythrina cristagalli	?	?	?	?	
Erythrospermum phytolaccoides	1	1	1	1	
Erythroxylum confusum	1	1	1	1	
Escallonia rubra	1	1	1	1	
Eschscholzia californica	1	1	1	1	

Eucalyptus lehmannii	1	1	1	1	
Euclea crispa	1	1	1	1	
Eucnide bartonioides	1	1	1	1	
Eucommia ulmoides	1	1	1	1	
Eucryphia lucida	1	1	1	1	
Euonymus americanus	?	?	1	1	Matthews & Endress 2005, Bot. J. Linn. Soc. 149: 129-194 (Euonymus latifolius).
Euphorbia epithymoides	1	1	1	1	
Euphronia guianensis	1	1	1	1	
Eupomatia bennettii	1	1	1	1	
Euptelea polyandra	0	0	0	0	
Eurya japonica	1	1	1	1	
Exacum affine	1	1	?	?	It is possible that the lowermost parts of the carpels are congenitally united, see Gopal Krishna and Puri 1962, Bot. Gaz. 124: 42-57.
Exbucklandia populnea	1	1	1	1	
Fagopyrum esculentum	1	1	1	1	
Fagus grandifolia	?	?	1	1	Sogo, Tobe, 2006, Amer. J. Bot. 93: 1748–1756 (Fagus)
Fallopia sachalinensis	?	?	1	1	we follow the view that the gynoecium of Polygonaceae has 2-3 carpels
Fendlera rupicola	?	?	?	?	
Ficus carica	?	?	1	1	the gynoecium is pseudomonomerous; development: Beck & Lord 1988, Amer. J. Bot. 75: 1904-1912.
Flacourtia indica	1	1	1	1	
Flagellaria indica	1	1	1	1	
Floerkea proserpinacoides	?	?	?	?	
Forgesia racemosa	1	1	1	1	
Forstera bidwillii	?	?	?	?	
Fouquieria splendens	1	1	1	1	
Frankenia pulverulenta	?	?	1	1	Ronse De Craene 2010, Floral Diagrams (F. laevis)
Fuchsia procumbens	1	1	1	1	
Gaiadendron punctatum	1	1	1	1	
Galax urceolata	1	1	1	1	
Galbulimima belgraveana	?	?	0	0	Igersheim and Endress 1997, Bot. J. Linn. Soc. 124: 213-271.

Galearia filiformis	1	1	1	1	
Galium aparine	1	1	1	1	
Garcinia subelliptica	1	1	1	1	
Garrya elliptica	1	1	1	1	
Geissoloma marginatum	1	1	1	1	
Gelsemium sempervirens	1	1	?	?	insufficient data
Gentiana saponaria	1	1	0	0	carpel fusion is postgenital in Gentianaceae, e.g., Albert & Struwe 2002, Gentianaceae: systematics and natural history.
Geranium sanguineum	1	1	1	1	
Gerbera jamesonii	?	?	1	1	we follow the interpretation of the gynoecium as dimerous in Asteraceae
Gerrardina foliosa	1	1	?	?	insufficient data
Gilia capitata	1	1	1	1	
Gisekia africana	0	0	?	?	Hoffmann 1994, in Behnke et al. (eds.), Caryophyllales
Gladiolus buckerveldii	1	1	1	1	
Glaucidium palmatum	0	0	?	?	the two carpels are basally slightly connate, developmental data not available
Glycine max	?	?	?	?	
Gomortega keule	1	1	1	1	
Gomphandra javanica	1	1	1	1	
Gonocaryum litorale	1	1	1	1	
Goodenia ovata	?	?	1	1	we accept the syncarpous interpretation for the gynoecium of Goodeniaceae
Gossypium hirsutum	1	1	1	1	
Goupia glabra	1	1	1	1	
Grevillea robusta	?	?	?	?	
Greyia radlkoferi	1	1	1	1	
Griselinia littoralis	1	1	1	1	
Grubbia tomentosa	?	?	?	?	
Guaiacum sanctum	1	1	1	1	
Guamatela tuerckheimii	?	?	?	?	
Guizotia abyssinica	1	1	1	1	
Gunnera hamiltonii	1	1	1	1	

Gymnosporia senegalensis	?	?	?	?	
Gyrocarpus americanus	?	?	?	?	
Gyrostemon tepperi	?	?	?	?	
Halesia carolina	1	1	1	1	
Halleria lucida	1	1	1	1	
Halophytum ameghinoi	1	1	1	1	
Haloragis aspera	1	1	1	1	
Hamamelis virginiana	?	?	1	1	Mione & Bogle 1990, Amer. J. Bot. 77: 77-91.
Hedera helix	1	1	1	1	
Hedycarya arborea	0	0	0	0	
Hedyosmum arborescens	?	?	?	?	
Heisteria parvifolia	1	1	1	1	
Helianthemum grandiflorum	1	1	1	1	
Helianthus annuus	1	1	1	1	
Helwingia japonica	1	1	1	1	
Heptacodium miconioides	?	?	1	1	the ovary is 3-locular (Qiner & Landrein in Flora of China, vol. 19)
Hernandia ovigera	?	?	?	?	
Heteromorpha trifoliata	?	?	1	1	the gynoecium of Apiaceae is bicarpellate, syncarpous
Heteropyxis natalensis	1	1	1	1	
Heuchera micrantha	1	1	1	1	
Hevea brasiliensis	?	?	?	?	
Heywoodia lucens	?	?	?	?	
Hibbertia volubilis	0	0	0	0	
Hirtella bicornis	?	?	?	?	
Homalanthus populneus	1	1	1	1	
Hordeum bulbosum	?	?	1	1	we follow the pseudomonomerous interpretation of grass gynoecium
Hortonia floribunda	0	0	0	0	

Houttuynia cordata	1	1	1	1	
Hua gabonii	1	1	1	1	
Hugonia platysepala	1	1	1	1	
Humiria balsamifera	1	1	1	1	
Humulus lupulus	?	?	1	1	Leme & Teixeira 2016, European Microscopy Congress 2016: Proceedings
Hura crepitans	1	1	1	1	
Hybanthus concolor	1	1	1	1	
Hydnocarpus heterophylla	1	1	1	1	
Hydrangea macrophylla	?	?	1	1	Roels et al. 1997, Nord. J. Bot. 17: 235-254 (H. petiolaris)
Hydrastis canadensis	0	0	0	0	
Hydrocharis dubia	1	1	1	1	
Hydrocotyle vulgaris	1	1	1	1	
Hydrolea ovata	1	1	1	1	
Hydrophyllum capitatum	?	?	1	1	ovary 1-locular with 2 parietal placenta (Hydrophyllum, Wilson 1960, Journal of the Arnold Arboretum, 41: 197-212)
Hydrostachys multifida	1	1	1	1	
Hymenanthera alpina	1	1	1	1	
Hypecoum imberbe	1	1	1	1	
Hypericum perforatum	1	1	1	1	
Hypertelis spergulacea	0	0	?	?	not enough data
Hypoxis hemerocallidea	1	1	1	1	
Icacina mannii	?	?	?	?	
Idesia polycarpa	1	1	1	1	
Idiospermum australiense	0	0	0	0	
Ilex cornuta	1	1	1	1	
Illicium floridanum	?	?	0	0	Endress 2001, Int. J. Plant Sci. 162: 1111–1140.
Impatiens repens	1	1	1	1	
Indigofera heterantha	?	?	?	?	
Ipomoea alba	?	?	1	1	Kajita & Nishino 2009, J. Japan. Soc. Hort. Sci. 78: 369–380 (I. nil).
Iris missouriensis	1	1	1	1	

Irvingbaileya australis	1	1	1	1	
Irvingia malayana	1	1	1	1	
Itea virginica	1	1	?	?	not enough data
Ixerba brexioides	1	1	1	1	
Ixiolirion tataricum	1	1	1	1	
Japonolirion osense	1	1	0	0	Remizowa et al. 2006, Pl. Syst. Evol. 258: 183–209.
Jasminum simplicifolium	1	1	1	1	
Joinvillea plicata	1	1	1	1	
Juglans mandshurica	?	?	1	1	Lin et al. 2016, Bot. J. Linn. Soc. 181, 279–293 (J. regia).
Juncus effusus	?	?	1	1	Oriani et al. 2012, Flora 207: 334– 340.
Junellia succulentifolia	?	?	?	?	
Justicia americana	1	1	1	1	
Kadsura japonica	0	0	0	0	
Kalanchoe daigremontiana	1	1	1	1	
Kiggelaria africana	1	1	1	1	
Kingdonia uniflora	0	0	0	0	
Klainedoxa gabonensis	1	1	1	1	
Koeberlinia spinosa	1	1	1	1	
Kolkwitzia amabilis	1	1	1	1	
Krameria ixine	?	?	1	1	Simpson 1982, Taxon 31: 517-528 (Krameria)
Lachnostylis bilocularis	1	1	1	1	
Lacistema aggregatum	1	1	1	1	
Lactoris fernandeziana	0	0	0	0	
Lactuca sativa	?	?	1	1	we follow the interpretation of the gynoecium in Asteraceae as bicarpellate
Lamium amplexicaule	?	?	1	1	Lord 1982, Bot. Gaz. 143: 63-72.
Lampranthus blandus	1	1	1	1	
Lapageria rosea	1	1	1	1	
Lardizabala biternata	0	0	0	0	
Larrea tridentata	?	?	?	?	

Lasiocroton bahamensis	1	1	1	1	
Laurus nobilis	?	?	?	?	
Leea guineense	1	1	1	1	
Lemna minor	?	?	?	?	
Leonia glycycarpa	1	1	1	1	
Lepidobotrys staudtii	?	?	?	?	
Leycesteria formosa	1	1	1	1	
Lilium superbum	?	?	1	1	Greller & Matzke 1970, Bot. Gaz. 131: 304-311 (L. tigrinum).
Limeum africanum	1	1	1	1	
Limonium arborescens	1	1	1	1	
Linnaea borealis	?	?	1	1	Wilkinson 1948, Amer. J. Bot. 35: 365-371.
Linum perenne	1	1	1	1	
Liquidambar styraciflua	1	1	1	1	
Liriodendron chinense	0	0	0	0	
Lissocarpa benthamii	1	1	1	1	
Lobelia angulata	?	?	1	1	there are 2 carpels in Lobelia
Lomandra longifolia	1	1	1	1	
Lonicera japonica	1	1	1	1	
Lophopyxis maingayi	1	1	1	1	
Lotus corniculatus	?	?	?	?	
Lozania pittieri	1	1	1	1	
Luculia gratissima	1	1	1	1	
Lunania parviflora	1	1	1	1	
Luxemburgia octandra	1	1	1	1	
Lythrum salicaria	1	1	1	1	
Mackinlaya confusa	?	?	1	1	gynoecium is syncarpous in Apiales
Maesa tenera	1	1	1	1	
Magnolia tripetala	?	?	0	0	Xu & Rudall 2006, Plant Syst. Evol. 258: 1-15 (Magnoila).
Mahonia bealei	?	?	?	?	
Malesherbia linearifolia	1	1	1	1	
Malpighia emarginata	?	?	?	?	
Mammea americana	1	1	1	1	

Manihot esculenta	?	?	?	?	
Manilkara zapota	1	1	1	1	
Maranta cristata	?	?	1	1	Andersson 1998, in Kubitzki, Fam. Gen. Vascular Plants, 4
Marathrum rubrum	1	1	1	1	
Marcgravia trinitatis	1	1	1	1	
Martynia annua	1	1	1	1	
Mauloutchia chapelieri	?	?	?	?	
Mayaca fluviatilis	1	1	1	1	
Mazus pumilus	1	1	1	1	
Medicago sativa	?	?	?	?	
Medusagyne oppositifolia	1	1	1	1	
Melanophylla alnifolia	1	1	1	1	
Melianthus major	1	1	1	1	
Meliosma veitchiorum	1	1	1	1	
Menispermum canadense	?	?	0	0	Endress and Igersheim 1999, Bot. J. Linn. Soc. 130: 305–393.
Mentzelia lindleyi	1	1	1	1	
Menyanthes trifoliata	1	1	1	1	
Mesua larnachiana	1	1	1	1	
Metanarthecium luteoviride	1	1	1	1	
Metrosideros nervulosa	1	1	1	1	
Micrantheum hexandrum	1	1	1	1	
Microdesmis puberula	1	1	1	1	
Mimosa polycarpa	?	?	0	0	http://www.plantillustrations.org/illustration.php?id illustration=6619
Minquartia guianensis	1	1	1	1	
Mirabilis jalapa	?	?	?	?	
Misodendrum linearifolium	1	1	1	1	
Mitchella repens	1	1	1	1	
Molineria capitulata	1	1	1	1	
Mollugo verticillata	1	1	1	1	

Montinia caryophyllacea	1	1	1	1	
Morina longifolia	1	1	1	1	
Morus indica	?	?	1	1	there are two stigmas in Morus
Moschopsis rosulata	?	?	?	?	
Moultonianthus leembruggianus	1	1	1	1	
Mouriri cyphocarpa	1	1	1	1	
Musa acuminata	1	1	1	1	
Myodocarpus fraxinifolius	?	?	1	1	Erbar and Leins 2010, Plant Div. Evol. Vol. 128: 269–295 (Myodocarpus vieillardii).
Myoporum mauritianum	1	1	1	1	
Myrica cerifera	1	1	1	1	
Myrica gale	1	1	1	1	
Myriophyllum sibiricum	?	?	1	1	fruit 4-loculed (Flora of China)
Myristica fragrans	?	?	?	?	
Myrothamnus flabellifolia	1	1	1	1	
Myrtus communis	1	1	1	1	
Najas minor	?	?	?	?	
Nandina domestica	?	?	?	?	
Nardostachys chinensis	1	1	1	1	
Nelumbo lutea	0	0	0	0	
Neoscortechinia kingii	1	1	1	1	
Nepenthes alata	1	1	1	1	
Nephrophyllidium cristagalli	1	1	1	1	
Nerium oleander	1	1	0	0	Thomas, Dave, 1991, Feddes Repertorium 102: 399-407
Neurada procumbens	1	1	1	1	
Nicotiana tabacum	1	1	1	1	
Nitraria retusa	1	1	1	1	
Nolana humifusa	1	1	1	1	
Nothofagus antarctica	1	1	1	1	
Nothoscordum bivalve	1	1	1	1	
Nuphar advena	1	1	1	1	
Nymphaea odorata	1	1	1	1	

Nymphoides peltata	1	1	1	1	
Nyssa ogeche	?	?	?	?	
Ochanostachys amentacea	1	1	1	1	
Ochna multiflora	?	?	?	?	
Ochroma pyramidale	1	1	1	1	
Ochthocosmus longipedicellatus	?	?	?	?	
Oenothera parviflora	1	1	1	1	
Olea europaea	?	?	1	1	Cuevas et al. 1999, Proc. 3rd. Intern. ISHS Symp. on olve growing, Acta Hort. 474: 293-296.
Olinia ventosa	1	1	1	1	
Omphalea diandra	1	1	1	1	
Oncidium excavatum	1	1	1	1	
Oncotheca balansae	1	1	1	1	
Opilia amentacea	?	?	?	?	
Opuntia microdasys	?	?	1	1	Leins and Erbar, 2010, Flower and fruit: morphology, ontogeny, phylogeny, function and ecology. Stuttgart: Schweizerbart: 112-113 (O. Leuctricha)
Orontium aquaticum	?	?	?	?	
Oryza sativa	?	?	1	1	we follow the pseudomonomerous interpretation of grass gynoecium
Osyris lanceolata	?	?	?	?	
Oxalis dillenii	1	1	1	1	
Pachysandra procumbens	?	?	1	1	proximal parts of carpels are congenitally united in Pachysandra spp. , von Balthazar and Enress 2002, Bot. J. Linn. Soc. 140: 193–228
Paeonia californica	0	0	0	0	
Panax quinquefolius	1	1	1	1	
Panda oleosa	1	1	1	1	
Pangium edule	1	1	1	1	
Paracryphia alticola	1	1	1	1	
Paradrypetes subintegrifolia	?	?	?	?	
Parnassia palustris	1	1	1	1	
Paropsia madagascariensis	1	1	1	1	
Passiflora biflora	1	1	1	1	
Patrinia triloba	1	1	1	1	

Paulownia tomentosa	1	1	1	1	
Paxistima canbyi	?	?	?	?	
Pedicularis foliosa	1	1	1	1	
Pelargonium cotyledonis	1	1	1	1	
Peltanthera floribunda	1	1	1	1	
Pennantia corymbosa	1	1	1	1	
Pentadiplandra brazzeana	1	1	1	1	
Pentaphragma ellipticum	?	?	?	?	
Penthorum sedoides	1	1	1	1	
Peperomia caliginigaudens	?	?	?	?	
Pera bicolor	1	1	1	1	
Pereskia aculeata	1	1	1	1	
Peridiscus lucidus	1	1	1	1	
Perrottetia ovata	1	1	1	1	
Petalonyx nitidus	?	?	?	?	
Petalostigma pubescens	1	1	1	1	
Petrophile canescens	?	?	?	?	
Petrosavia sakuraii	?	?	1	1	M.V.Remizowa, pers. obs.
Petunia axillaris	?	?	1	1	Vandenbussche et al. 2009, Plant Cell 21: 2269–2283 (P. hybrida).
Peumus boldus	0	0	0	0	
Phalaenopsis aphrodite	1	1	1	1	
Phaulothamnus spinescens	1	1	1	1	
Phelline billardierei	1	1	1	1	
Phenakospermum guyannense	?	?	?	?	
Philadelphus lewisii	?	?	1	1	Roels et al. 1997, Nord. J. Bot. 17: 235-254 (P. purpurascens)
Philesia magellanica	?	?	1	1	Conran and Clifford in Kubitzki 1998
Philydrum lanuginosum	1	1	1	1	
Phlox longifolia	1	1	1	1	
Phoenix dactylifera	?	?	0	0	Rudall et al. 2011, Int. J. Plant Sci. 172: 674-690.
Photinia x fraseri	?	?	1	1	Sterling 1965, Amer. Jour. Bot. 52(9): 938-946 (Photinia)

Phryma leptostachya	1	1	1	1	
Phyllanthus flexuosus	?	?	?	?	
Phyllonoma laticuspis	1	1	1	1	
Physena madagascariensis	1	1	1	1	
Phytolacca americana	1	1	1	1	
Picramnia polyantha	1	1	?	?	developmental data are needed
Pilea cadierei	?	?	?	?	
Pimelodendron zoanthogyne	1	1	1	1	
Pinguicula moranensis	?	?	1	1	we follow the syncarpous interpretation of the gynoecium of Pinguicula
Piper betle	1	1	1	1	
Pisum sativum	?	?	?	?	
Pittosporum tobira	1	1	1	1	
Plagiopteron suaveolens	?	?	?	?	
Plantago lanceolata	1	1	1	1	
Platanus occidentalis	0	0	0	0	
Platysace lanceolata	?	?	1	1	the gynoecium of Apiaceae is bicarpellate, syncarpous
Platyspermation crassifolium	1	1	1	1	
Pleea tenuifolia	?	?	0	0	Utech 1978, Ann. Carnegie Mus. 47: 423–454.
Plocosperma buxifolium	1	1	1	1	
Plumbago auriculata	?	?	?	?	
Podocalyx loranthoides	1	1	1	1	
Podophyllum peltatum	?	?	?	?	
Podostemum ceratophyllum	1	1	1	1	
Pogonophora schomburgkiana	?	?	?	?	
Polemonium reptans	1	1	1	1	
Poliothyrsis sinensis	1	1	1	1	
Polygala pauciflora	?	?	1	1	Prenner 2004, Plant Syst. Evol. 249: 67–76 (Polygala myrtifolia).

Polyosma cunninghamii	?	?	?	?	
Polypremum procumbens	1	1	1	1	
Polyscias guilfoylei	?	?	1	1	Xiang and Lowry, Flora of China, Vol. 13
Pontederia cordata	?	?	1	1	fusion between carpels is mostly postgenital, but central part of the gynoecium in its basal portion is formed by continuous growth (see van Heel 1988, Blumea 33: 477-504; Strange et al. 2004, Bot. J. Linn. Soc. 144: 395–408).
Populus tremuloides	1	1	1	1	
Portulaca grandiflora	1	1	1	1	
Potamogeton berchtoldii	0	0	0	0	
Primula sieboldii	1	1	1	1	
Prockia crucis	1	1	1	1	
Prunus persica	?	?	?	?	
Pseudonemacladus oppositifolius	1	1	1	1	
Pseudopanax arboreus	1	1	1	1	
Pterocephalodes hookeri	1	1	1	1	
Pterostemon rotundifolius	1	1	1	1	
Puya raimondii	?	?	0	0	Sajo et al. 2004, Plant Syst. Evol. 247: 215-231
Qualea grandiflora	1	1	1	1	
Quercus rubra	?	?	1	1	Kaul 1985, Amer. J. Bot. 72: 1962-1977.
Quiina pteridophylla	?	?	?	?	
Quillaja saponaria	0	0	1	1	Bello et al. 2007, Ann. Bot. 100: 1491–1505.
Quintinia verdonii	1	1	1	1	
Ranunculus acris	?	?	0	0	Zhao et al. 2012, Plant. Syst. Evol. 298: 1057–1071 (Ranunculus).
Raphanus sativus	?	?	1	1	http://www.botany.hawaii.edu/faculty/carr/images/rap sat.jpg
Ravenala madagascariensis	1	1	1	1	
Reinwardtia indica	1	1	1	1	
Reseda alba	1	1	1	1	
Rhabdodendron amazonicum	?	?	?	?	
Rhamnus cathartica	1	1	1	1	

Rhizophora stylosa	?	?	1	1	Schwarzbach, 20014, in Kubitzki, Families and genera..., 11: 283-295 (Rhizophora).
Rhododendron hippophaeoides	1	1	1	1	
Rhodohypoxis milloides	1	1	1	1	
Rhodoleia championii	?	?	1	1	Endress, 1993, in Kubitzki, Families and genera..., 2: 322.
Rhus copallinum	?	?	1	1	Gallant et al. 1998, Int. J. Plant Sci. 159: 539-549 (Rhus hirta).
Rhynchoglossum notonianum	1	1	1	1	
Rhynchospora latifolia	?	?	1	1	Lucero et al. 2014, Int. J. Plant Sci. 175: 186-201 (Rhynchospora).
Ribes aureum	?	?	1	1	Yen 1936, Bot. Gaz. 98: 105-120.
Ricinus communis	?	?	?	?	
Rinorea pubiflora	1	1	1	1	
Rivina humilis	?	?	?	?	
Roridula gorgonias	1	1	1	1	
Roupala montana	?	?	?	?	
Rourea minor	0	0	0	0	
Roussea simplex	?	?	?	?	
Ruptiliocarpon caracolito	1	1	1	1	
Sabia swinhoei	1	1	1	1	
Saccharum officinarum	?	?	1	1	we follow the pseudomonomerous interpretation of grass gynoecium
Sacoglottis amazonica	1	1	1	1	
Salix reticulata	1	1	1	1	
Sambucus racemosa	1	1	1	1	
Sanicula gregaria	1	1	1	1	
Santalum album	1	1	1	1	
Sarcandra chloranthoides	?	?	?	?	
Sarcobatus vermiculatus	1	1	1	1	
Sargentodoxa cuneata	0	0	0	0	
Sarracenia purpurea	1	1	1	1	
Saruma henryi	1	1	1	1	
Sassafras albidum	?	?	?	?	
Saururus cernuus	1	1	1	1	
Sauvagesia erecta	1	1	1	1	
Saxifraga cernua	1	1	1	1	

Scabiosa columbaria	?	?	?	?	
Scaevola aemula	?	?	1	1	https://profiles.ala.org.au/opus/foa/profile/Scaevola%20aemula
Schefflera arboricola	?	?	1	1	Nuraliev et al. 2017. Evolutionary Floral Morphology of Araliaceae: A Case Study of the Asian Schefflera; MAKS Press: Moscow; ISBN 978-5-317-05663-6.
Schinus molle	1	1	1	1	
Schisandra chinensis	0	0	0	0	
Schoepfia schreberi	1	1	1	1	
Schotia brachypetala	?	?	?	?	
Scrophularia californica	1	1	1	1	
Scyphostegia borneensis	1	1	1	1	
Sedum rubrotinctum	?	?	?	?	
Sesamum indicum	1	1	1	1	
Simmondsia chinensis	1	1	1	1	
Sinadoxa corydalifolia	?	?	1	1	Backlund and Bittrich, 2016, in Kubitzki, Families and genera..., 14: 25.
Siparuna decipiens	0	0	0	0	
Siphonodon celastrineus	1	1	1	1	
Sloanea latifolia	1	1	1	1	
Smilax glauca	1	1	1	1	
Solanum dulcamara	1	1	1	1	
Soyauxia talbotii	1	1	1	1	
Sparganium eurycarpum	1	1	1	1	
Spathiostemon javensis	?	?	?	?	
Spathiphyllum wallisii	1	1	1	1	
Sphenoclea zeylanica	1	1	1	1	
Sphenostemon lobosporus	1	1	1	1	
Spigelia marilandica	1	1	?	?	developmental data are needed
Spinacia oleracea	1	1	1	1	
Spiraea betulifolia	0	0	0	0	

Stachyurus praecox	1	1	1	1	
Stackhousia minima	1	1	1	1	
Staphylea trifolia	1	1	1	1	
Stegnosperma halimifolium	1	1	1	1	
Stegolepis ligulata	1	1	1	1	
Stellaria media	?	?	1	1	Moeliono 1959, Acta Bot. Neerl. 8: 292-303.
Sterculia apetala	1	1	0	0	Bayer and Kubitzki 2003, in Kubitzki, Families and genera..., 5: 263.
Strasburgeria robusta	1	1	1	1	
Strelitzia reginae	?	?	1	1	Kronestedt and Walles 1986, Nord. J. Bot. 6: 307-320.
Strychnos nuxvomica	1	1	?	?	developmental data are needed
Stylidium graminifolium	1	1	1	1	
Stylobasium spathulatum	?	?	?	?	
Styrax officinalis	1	1	1	1	
Sullivantia oregana	1	1	1	1	
Suregada boiviniana	?	?	?	?	
Swietenia macrophylla	1	1	1	1	
Symphonia tanalensis	1	1	1	1	
Symphoricarpos albus	1	1	1	1	
Symplocos zizyphoides	1	1	1	1	
Syringa vulgaris	1	1	1	1	
Tacca chantrieri	?	?	1	1	Drenth 1972, Blumea 20: 367-406.
Tagetes erecta	1	1	1	1	
Takhtajania perrieri	1	1	1	1	
Talbotia elegans	1	1	1	1	
Talinum paniculatum	?	?	1	1	Veselova et al. 2012, Wulfenia 19: 107–129.
Tamarix chinensis	?	?	1	1	Yang and Gaskin, Flora of China, Vol. 13
Tapiscia sinensis	1	1	1	1	
Tapura guianensis	1	1	1	1	
Tasmannia insipida	?	?	?	?	
Tecophilaea cyanocrocus	1	1	1	1	

Terminalia catappa	1	1	1	1	
Ternstroemia stahlii	1	1	1	1	
Tetracarpaea tasmanica	0 & 1	0 & 1	0	0	Hills et al.1988, Amer. J. Bot. 75: 1687-1700. This species is problematic in scoring because of the nature of variation observed. We prefer scoring it as free-carpellate, because at least one carpel is normally free.
Tetracentron sinense	1	1	1	1	
Tetracera asiatica	?	?	?	?	
Tetracoccus dioicus	1	1	1	1	
Tetradoxa omeiensis	?	?	1	1	Backlund and Bittrich, 2016, in Kubitzki, Families and genera..., 14: 25.
Tetrameles nudiflora	1	1	1	1	
Tetramerista crassifolia	1	1	1	1	
Tetrapanax papyrifer	1	1	1	1	
Tetraplasandra hawaiensis	?	?	1	1	Costello and Motley 2004, Amer. J. Bot. 91: 644–655.
Tetrapterys tinifolia	?	?	?	?	
Tetrorchidium gabonense	?	?	?	?	
Thomandersia laurifolia	1	1	1	1	
Thottea tomentosa	?	?	1	1	Leins et al. 1988, Blumea 33: 357-370.
Thryallis latifolia	?	?	?	?	
Thunbergia alata	1	1	1	1	
Thymelaea hirsuta	?	?	?	?	
Tinospora sinensis	0	0	0	0	
Titanotrichum oldhamii	?	?	?	?	
Tofieldia calyculata	?	?	0	0	Leinfellner 1962, Österr. Bot. Zeitschr. 109: 1-17.
Torricellia tiliifolia	1	1	1	1	
Touroulia guianensis	1	1	1	1	
Tovaria pendula	1	1	1	1	
Trachycarpus fortunei	?	?	0	0	Rudall et al. 2011, Int. J. Plant Sci. 172: 674-690.
Tradescantia spathacea	1	1	1	1	

Tragopogon dubius	1	1	1	1	
Trichilia emetica	1	1	?	?	in the absence of developmental data, it is difficult to ensure that the fusion is congenital, as postgenital fusions are present in some related taxa
Triglochin maritima	1	1	1	1	
Trigonia nivea	1	1	1	1	
Trigonostemon verrucosus	1	1	1	1	
Trillium erectum	?	?	1	1	Takahashi 1994, J. Plant Res. 107 : 237-243 (Trillium)
Trimenia moorei	?	?	?	?	
Triosteum perfoliatum	1	1	1	1	
Triphyophyllum peltatum	1	1	1	1	
Triplostegia glandulifera	1	1	1	1	
Tripterygium wilfordii	?	?	?	?	
Trithuria submersa	?	?	?	?	
Trochodendron aralioides	1	1	1	1	
Tropaeolum tricolor	1	1	1	1	
Turnera ulmifolia	1	1	1	1	
Typha latifolia	?	?	?	?	
Urtica dioica	?	?	?	?	
Utricularia alpina	1	1	1	1	
Vaccinium uliginosum	1	1	1	1	
Vahlia capensis	1	1	1	1	
Valdivia gayana	1	1	1	1	
Valeriana officinalis	?	?	1	1	Weberling and Bittrich, 2016, in Kubitzki, Families and genera..., 14: 385-401 (Valeriana).
Valerianella locusta	1	1	1	1	
Vantanea guianensis	1	1	1	1	
Verbascum thapsus	1	1	1	1	
Veronica anagallisaquatica	?	?	1	1	Kampny at al. 1993, Amer. J. Bot. 80: 449-460 (V. chamaedrys).
Viburnum acerifolium	1	1	1	1	
Villarsia calthifolia	1	1	1	1	
Viola pubescens	1	1	1	1	
Vismia baccifera	1	1	1	1	

Vitis aestivalis	1	1	1	1	
Viviania marifolia	1	1	?	?	The group of families to which belongs this genus is insufficiently studied with respect to flower development
Vochysia guatemalensis	?	?	?	?	
Vriesea psittacina	?	?	?	?	
Weigela hortensis	?	?	1	1	Hofmann and Bittrich, 2016 in Kubitzki, Families and genera..., 14: 124 (Weigela).
Wittsteinia vacciniacea	?	?	?	?	
Xanthorhiza simplicissima	?	?	0	0	Tamura 1993 in Kubitzki, Families and genera..., 2: 581.
Xanthorrhoea resinosa	1	1	1	1	
Xanthosoma sagittifolium	?	?	?	?	
Xerophyta retinervis	1	1	1	1	
Xerosicyos danguyi	1	1	1	1	
Ximenia americana	?	?	?	?	
Xyris jupicai	1	1	1	1	
Yucca filamentosa	1	1	1	1	
Zabelia tyaihyonii	?	?	1	1	Hofmann and Bittrich, 2016, Families and genera..., 14: 128 (Zabelia).
Zea mays	?	?	1	1	we follow the pseudomonomerous interpretation of grass gynoecium
Zelkova serrata	?	?	1	1	Okamoto et al. 1992, Amer. J. Bot. 79: 921-927.
Zingiber gramineum	?	?	1	1	Larsen et al. 1998 in Kubitzki, Families and genera..., 4: 489 (Zingiber).
